# Isoflavonoids from *Iris albicans* as a carbon source to enhance the anti-aging potential of lactic acid bacteria-derived postbiotics

**DOI:** 10.1038/s41598-026-52823-x

**Published:** 2026-06-04

**Authors:** Ahmed Elbermawi, Mohamed Samir Darwish, Noha A. Abou-Zeid, Ahmed Awad Zaki, Asmaa A. Elawady, Yhiya Amen

**Affiliations:** 1 Department of Pharmacognosy, Faculty of Pharmacy, Mansoura University, 35516 Mansoura, Egypt; 2 Dairy Department, Faculty of Agriculture, Mansoura University, Mansoura, 35516 Egypt; 3 Veterinary Medicine Directorate, Mansoura, 35516 Egypt

**Keywords:** Antiglycation, Gerobiotics, Isoflavonoids, Prebiotics, Postbiotics and Probiotics, Biochemistry, Health care, Microbiology

## Abstract

Dietary strategies that limit protein glycation can help slow aging and prevent age‑related diseases by reducing the accumulation of advanced glycation end products (AGEs). This study evaluated the antioxidant, antiglycation, and prebiotic potential of postbiotics produced by lactic acid bacteria using *Iris albicans* extract and its major isoflavonoids as carbon sources. Antioxidant activity (DPPH, ABTS, FRAP), prebiotic potential (A_preb_ activity scores), and antiglycation effects were assessed via fructosamine formation, oxidative stress markers, protein aggregation, and AGE‑RAGE inhibition assays. Among all tested postbiotics, *Limosilactobacillus reuteri* MSD37 showed the highest antioxidant and antiglycation activities, especially when cultured with *I. albicans* extract or irilone. This postbiotic effectively preserved thiol groups, reduced protein oxidation, and inhibited AGE‑RAGE interactions. Moreover, *L. reuteri* MSD37 postbiotic enriched with *I. albicans* extract exhibited notably high prebiotic activity toward probiotic strains. Overall, *I. albicans* represents a promising carbon source for producing anti‑aging postbiotics, highlighting the potential of *L. reuteri* MSD37 as a gerobiotic candidate for promoting healthy aging.

**Supplementary Information:**

The online version contains supplementary material available at 10.1038/s41598-026-52823-x.

## Introduction

Aging is commonly seen as a gradual decline in physiological function, potentially driven by biological processes influenced by reactive carbonyl species (RCS) and reactive oxygen species (ROS)^1^. Protein glycation induced by RCS and the resulting buildup of advanced glycation end products (AGEs) during aging have garnered significant attention, as these processes are considered a critical link between aging and various human diseases, including neurodegenerative disorders, diabetes, and atherosclerosis symptoms^2^. One of the most effective strategies for delaying the onset and progression of aging-related diseases is exploring health-promoting dietary approaches that can prevent RCS-induced glycation and the production of AGEs. Most AGEs formed in the body occur through glycation processes, which are heavily influenced by food intake. Along with the metabolic reactions of the human body, glucose concentrations are directly related to the amounts of AGEs formed^3^. The interaction of AGEs with their receptor (RAGE) activates multiple intracellular signaling pathways and gene expressions. This leads to pro-inflammatory chemicals secretion, which generates free radicals and contributes to other pathologic conditions associated with diabetes^4^.

An example of a possible approach to managing and preventing AGE-related disorders consists of feeding individuals with food that is anti-glycated. For example, phenolic components in citrus fruits have been shown to inhibit AGE formation^5^. The anti-glycation properties of isoflavones from legumes have also been proven to be efficient in vitro^6^. The human gastrointestinal tract frequently produces anti-AGE compounds through enzymatic hydrolysis or microbial fermentation^3^. Therefore, focusing on certain probiotics or their by-products (postbiotics) that have anti-AGEs activity may be a viable disease preventive tactic^3^.

Regulation of positive balance of microbiota through the application of postbiotics, which in one way or another support health in hosts, is a new technique in the field because postbiotics are a more recent member of the biotics family. Since postbiotics are created during fermentation or the metabolic activity of probiotics in the gut, they are sometimes thought of as byproducts or effects of probiotics. Many of the health advantages of probiotics are mediated by these non-viable microbial components, which include metabolites, enzymes, and cell fragments. Probiotics produce active molecules called postbiotics, which essentially connect their existence to noticeable physiological effects on the host^7^. Postbiotics made from different probiotic strains have been shown in several studies to have strong antioxidant properties^8^. Furthermore, by neutralizing glycation agents or preventing functional groups in proteins from interacting with them, these postbiotics efficiently prevent protein glycation^3,9^. Utilizing plant extract or polyphenol chemicals as a carbon source for probiotic cultures can greatly increase both their metabolic activity and the postbiotics’ bioactive qualities^10–13^. These findings underpin the emerging field of gerobiotics, which explores the role of probiotics and their derivatives in modulating aging processes^14^. Gerobiotics aim to improve health span by targeting key aging mechanisms such as oxidative stress and inflammation, highlighting the therapeutic potential of tailored probiotic and postbiotic interventions^15^.

In ancient and modern medicine, the genus *Iris* holds great promise on account of its varying phytochemical compositions. For many thousands of years, diverse civilizations have used the plant to treat many diseases among peoples^16^. In Traditional Chinese Medicine, the rhizomes of *Iris confusa* were used for the treatment of acute bronchitis and tonsillitis^17^ The rhizomes of *Iris pseudacorus* were used in the traditional medicine of Ireland and Britain in the treatment of colds, toothaches, and throat inflammations^18^. 2`, 5-dihydroxy-6, 7-methylenedioxy, a very effective anti-glycating agent isolated and purified from *Iris loczyi* has therapeutic potential for treating complications arising from late-stage diabetes^19^. Several studies have also been done on the polyphenol compounds of *Iris* rhizomes, specifically *I. germanica* and *I. pallida*, including the isolation of many flavonoids and isoflavonoids^20^.

Previous research has investigated the antimicrobial, antioxidant, and cytotoxic activities of *I. albicans*^21,22^. These studies demonstrated that *I. albicans* exhibits significant ability to combat microbial pathogens, scavenge free radicals, and exert cytotoxic effects, supporting its potential for therapeutic applications.

Using bioactive molecules as carbon sources to tailor postbiotics represents an emerging and underexplored strategy for enhancing their functional properties. Building on this concept, the present study investigates how Iris albicans extract and its major isoflavonoids influence the production and bioactivity of lactic acid bacteria‑derived postbiotics. Specifically, it evaluates their in vitro antioxidant, antiglycation, and prebiotic activities to identify functionally tailored postbiotics with potential “gerobiotic” properties that support healthy aging.

## Materials and methods

### Plant material

*Iris albicans* L. (Iridaceae) was collected during the flowering stage in April 2020 from Kafr El-Sheikh city, Egypt, 31.0972° N, longitude: 30.9474° E. The fresh rhizomes were separated and dried at 30 °C and 41% relative humidity. A voucher specimen [Ir-09] has been maintained in the Pharmacognosy Department Herbarium, Mansoura University.

### Chemicals

All chemicals and reagents were purchased from Sigma-Aldrich Corporation (St. Louis, MO, USA) (see Supplemental for details).

### Culture media and strains

Bacterial strains (*Limosilactobacillus fermentum* MSD24, *Lacticaseibacillus casei* MSD21, *Limosilactobacillus reuteri* MSD37, *Lactiplantibacillus plantarum* MSD74, *Lacticaseibacillus rhamnosus* ML57, *Lacticaseibacillus paracasei* MSD108 and *Escherichia coli* K12) were provided by the microbiology laboratory’s culture collection at the Dairy Department, Faculty of Agriculture, Mansoura University, Mansoura, Egypt. The bacterial culture media, including de Man, Rogosa, and Sharpe (MRS) broth, MRS agar, nutrient agar, and nutrient broth, were obtained from Thermo Fisher Scientific in Cairo, Egypt. The API ZYM test kit was purchased from bioMérieux SA (Marcy l’Etoile, France). (see supplemental for details). In the present study, six lactic acid bacteria (LAB) strains with documented probiotic potential were included overall. Among them, three strains (*L. casei* MSD21, *L. fermentum* MSD24, and *L.reuteri* MSD37) were used as postbiotic‑producing strains. In comparison, three additional strains (*L.plantarum* MSD74, *L. rhamnosus* ML57, and *L. paracasei* MSD108) served as indicator probiotics in the evaluation of the prebiotic activity score (A_preb_) of the postbiotics generated from MSD21, MSD24, and MSD37.

The decision to focus on only MSD21, MSD24, and MSD37 for postbiotic production was based on preliminary screening experiments, in which these three isolates showed superior growth performance when glucose was replaced by the isoflavonoid compounds (1–7) or the IR extract as sole carbon sources, compared with the other LAB strains (MSD74, ML57, and MSD108). On this basis, MSD21, MSD24, and MSD37 were selected as the most suitable producers for detailed postbiotic characterization, whereas MSD74, ML57, and MSD108 were retained as indicator strains for assessing the prebiotic activity of the resulting postbiotics.

### General procedures

^1^H and ^13^C-NMR spectra were generated on a Bruker DRX 600 NMR spectrometer (Bruker Daltonics INC., MA, USA) (see supplemental for details).

### Extraction and Isolation

The powdered rhizomes of *I. albicans* L. (305 g) were extracted with 70% methanol (6 × 2 L) at a temperature ranging from 20 °C to 25 °C to get a dark brown residue (17.6 g), referred to as the IR extract. The total alcoholic extract was subjected to Vacuum Liquid Chromatography (VLC) over normal silica gel using chloroform (CHCl_3_) with increasing proportions of methanol (MeOH) to obtain four fractions. The fractions were purified using different chromatographic techniques to obtain compounds **1**–**7** (see the supplemental for details).

### Preparation of MRS without glucose

The MRS medium was prepared using the following ingredients (g/L): peptone 10.0, beef extract 8.0, yeast extract 5.0, polysorbate 80 1.0, ammonium citrate 2.0, sodium acetate (anhydrous) 3.0, magnesium sulfate 0.1, manganese sulfate 0.05, and dipotassium phosphate 2.0, without glucose. At 25 °C, the final pH was adjusted to 6.2 ± 0.2. A concentration of 2 mg/mL of each isoflavonoid compound (**1**–**7**) or the *I. albicans* root (IR) extract was added separately to the medium, while the positive control received an identical amount of dextrose. This concentration (2 mg/mL) was selected based on preliminary experiments showing that lower levels produced minimal effects on prebiotic activity. In contrast, higher levels did not further enhance activity, were more difficult to dissolve, and did not ensure optimal diffusion. At 2 mg/mL, the extract and individual compounds were soluble, stable in the medium, and provided reliable experimental conditions. Finally, 29.2 g of the dehydrated medium were dissolved in 1 L of deionized or distilled water, heated to boiling with stirring until fully dissolved, dispensed into final containers, and sterilized at 121 °C for 15 min.

### Preparation of postbiotics from lactic acid bacteria

Three different lactic acid bacteria strains (*Limosilactobacillus fermentum* MSD24, *Lacticaseibacillus casei* MSD21, *Limosilactobacillus reuteri* MSD37) were activated by culturing them overnight in MRS broth at 37 °C for 16–18 h, with fifth-generation strains being used for further experiments. The bacteria were then grown in MRS broth without glucose, supplemented with individual isoflavonoid compounds (**1**–**7**) or IR extract, and incubated at 37 °C for 18 h. The bacterial suspensions were heated at 70 °C for 45 min, centrifuged at 5,000 g for 15 min at 5 °C, and filter-sterilized using a 0.45 μm Millipore filter. The supernatant (postbiotic) was finally freeze-dried at − 50 °C for 48 h, and the required concentration was dissolved in phosphate-buffered saline (PBS, pH 7.2, 50 mM). The resulting postbiotics were named MSD-21, MSD-24, and MSD-37 according to the producing strain. Postbiotics were stored at −20 °C in sterile, airtight containers to preserve their bioactivity for subsequent analyses and applications.

### Antioxidant activity

#### Measurement of DPPH radical scavenging activity

The radical scavenging activity of postbiotics was evaluated using a slightly modified version of the method described by Oh, et al^23^. Besides the 100 µL of 200 µM 2,2-Diphenyl-1-Picrylhydrazyl (DPPH) in ethanol, 100 µL of the postbiotic (100 µg/mL) was added. After a vigorous stirring, the mixture was allowed to incubate at 37 °C in the dark for 30 min. The absorbance of the solution was then measured at 517 nm (OD, Optical Density) using spectrophotometry. Ascorbic acid (25 µg/mL) was used as a positive control.

#### Evaluation of ABTS radical scavenging activity

The postbiotics were assayed for 2,2’-Azinobis(3-ethylbenzothiazoline-6-sulfonic acid) (ABTS) radical scavenging activity according to the method of Oh, et al^23^. A solution containing 2.45 mM potassium persulfate and 7 mM ABTS was initially diluted with deionized water to about 1.4 absorbance, measured at 734 nm. Twenty microliters of the postbiotic (100 µg/mL) were added to 180 µL of this diluted ABTS solution for agitation, followed by 6 min of dark incubation at 25 °C. Ascorbic acid (15 µg/mL) was used as a positive control in absorbance measurement at 734 nm. For both DPPH and ABTS assays, the percentage of radical scavenging activity was calculated using the same equation:

1 $${\text{Radical scavenging activity}}\:\left({\%}\right)\hspace{0.17em}=\hspace{0.17em}[OD\:\left(Positive\:control\right)-OD\:\left(Sample\right)/\:OD\:\left(Positive\:control\right)]\:\times\:100$$

#### Ferric-reducing antioxidant power (FRAP) assay

The reducing ability of postbiotics was assessed using the ferric-reducing antioxidant power (FRAP) test, as reported by Oh, et al^23^. Synthesis of the FRAP reagent involved mixing 10 mM 2,4,6-Tripyridyl-s-triazine (TPTZ), 0.3 M sodium acetate buffer (pH 3.6) in 40 mM HCl, and 20 mM FeCl_3_·6H_2_O at a volume ratio of 10:1:1. This mixture was incubated at 37 °C for 30 min after adding 180 µL of the freshly made FRAP reagent with 6 µL of postbiotic (100 µg/mL). Ascorbic acid (100 µg/mL) was used as a positive control. The values for FRAP were determined by the standard curve of Fe_2_SO_4_.

### Determination of total phenolic compounds

The total phenolic components of postbiotics were assessed using an assay following the method described by Oh, et al^24^. To obtain the total phenolic content (TPC), calculated into micrograms of gallic acid equivalent (GAE) per milliliter, regression of known Gallic acid standards was performed against samples.

### In vitro antiglycation assay with BSA-fructose model

Bovine serum albumin (BSA) was glycated with slight modifications of the method of Grzegorczyk-Karolak, et al^25^. Briefly, 10 mg/mL BSA solution (500 µL) was incubated for seven days at 37 °C with 460 µL of 500-mM fructose in 100 mM phosphate-buffered saline (pH 7.4) containing 0.02% sodium azide. Before incubation, 40 µL of various postbiotics (MSD-21, MSD-24, and MSD-37) at a concentration of 2 mg/mL was added to the mixtures. The glycated BSA was then assessed by measuring fluorescent intensity at an excitation wavelength of 335 nm and an emission wavelength of 385 nm. The percentage of inhibition was determined using the following formula:

2 $$\begin{aligned}\:\%\:AGE\:Inhibition\:=\:(F\:control\:-\:F\:control~~blank)\\-\:(F\:postbiotic\:-\:F\:postbiotic~~blank)\:/\:(F\:control\:-\:F\:control~~blank)\:\times\:100\end{aligned}$$

where (F control – F control blank) is the difference between the fluorescent intensity of BSA incubated with or without fructose, and (F postbiotic– F postbiotic blank) is the difference between the fluorescent intensity of BSA and sugars incubated with or without postbiotic. Aminoguanidine (AG) was prepared by dissolving aminoguanidine hydrochloride in distilled water to a final concentration of 0.50 mg/mL, vortexing until fully dissolved, and volume-adjusting with distilled water. The solution was freshly prepared and sterilized using a 0.22 μm membrane filter before use.

#### Early glycation stage

##### Amadori product measurements

The concentration of fructosamine, an Amadori product, in glycated albumin samples (with and without postbiotics) was measured after 7 days using the nitroblue tetrazolium (NBT) assay, as described by Ardestani and Yazdanparast^26^. Briefly, 90 µL of 0.5 mM NBT in 100 mM carbonate buffer (pH 10.4) was incubated with 10 µL of glycated BSA for 15 min at 37 °C, and absorbance was recorded at 530 nm. Fructosamine levels were calculated from a standard curve of 1‑deoxy‑1‑morpholinofructose (1‑DMF). AG was used as a positive control.

##### Measurement of ∑- amino groups quantitatively

The 2,4,6-trinitrobenzene sulfonic acid (TNBSA) test is a rapid and precise method for determining free amino groups. Brightly colored derivatives formed by primary amines and TNBSA can be used in spectrophotometry at 335 nm. The procedure includes the mixing of 0.5 ml from each glycated BSA sample with 0.25 ml of a 0.01% (w/v) solution of TNBSA before incubating for two hours at 37 °C. Absorbance at 335 nm is then measured after adding 0.25 ml of 10% sodium lauryl sulfate (SDS) and 0.125 ml of 1 N HCl to each sample^3^. A standard curve constructed from lysine at a known concentration range was utilized to quantitatively determine the concentration of free amino groups (expressed as nmol/mg protein) in the sample. AG was used as the positive control.

#### Oxidation stage

##### Determination of the protein’s carbonyl content

The method by Meenatchi, et al^27^. was employed with some variations to measure the protein carbonyl levels. In short, 0.10 mL of glycated BSA was incubated with 0.40 mL of 10 mM 2,4-dinitrophenylhydrazine (DNPH) in 2.5 M HCl at room temperature for 60 min and then precipitated with 0.50 mL of 20% (w/v) trichloroacetic acid (TCA). It was then placed on ice for five minutes and centrifuged at 10,000 g for ten minutes at 4 °C. A 1:1 (v/v) ethanol: ethyl acetate solution (1 mL) was used to wash the pellet three times. The last pellet was dissolved in 0.25 mL of 6 M guanidine hydrochloride. At 370 nm, the absorbance was measured. The absorbance coefficient of 22,000 M^− 1^ cm^− 1^ was used to quantify the number of carbonyls in proteins. nmol carbonyls/mg protein was the unit of measurement used for the results. AG was used as the positive control.

##### Measurement of the thiol group

Using 5,5`-dithiobis (2-nitrobenzoic acid) (DTNB), Ellman’s assay was used to ascertain the amounts of free thiols (sulfhydryl groups) in each glycated BSA sample^28^. To put it briefly, 100 µL of glycated BSA and 50 µL of freshly prepared DTNB (3 mM) in 0.2 M phosphate, pH 7.4, were well mixed, and the combination was then incubated for 15 min at 37 °C. The quantity of thiol groups in each sample was determined by measuring the absorbance at 412 nm and using the standard curve, which was produced using various dosages of L-cysteine (Sigma). The results are expressed as the number of free SH groups per milligram of BSA. AG was used as the positive control.

#### Cross-linking stage

##### Protein aggregation measurement

With a few minor modifications, the approach of Meenatchi, et al^27^. was used to evaluate amyloid cross-β-structure, a frequent biomarker of protein aggregation, using thioflavin T, a dye unique to amyloid cross-β-structures. A 32 µM solution of thioflavin T was made in a 50 mM, pH 8.5 glycine-NaOH buffer. Three mL of the thioflavin T solution was mixed with the glycated samples (100 µL, as detailed in Sect. 2.10.1) and incubated for 60 min. Fluorescence was measured at an emission wavelength of 485 nm and an excitation wavelength of 435 nm (slit width: 10 nm), using appropriate blanks without thioflavin T. The results were reported in arbitrary units (AU). AG is used as a positive control.

#### Signaling stage

##### Assessment of glycated protein–RAGE interaction

The CircuLex AGE-RAGE in vitro Binding Assay Kit (No. CY-8151, MBL Medical) was utilized to evaluate the inhibitory effects of MSD-21, MSD-24, and MSD-37 postbiotics, with or without isoflavonoid compounds, on RAGE binding—a critical biological process linked to the development and progression of various diseases^3^. The procedure was performed following the manufacturer’s protocol^3^. AG is used as a positive control.

### Prebiotic activity score (A_preb_) of postbiotics

The impact of different types of postbiotic enriched with compounds (**1**–**7**) and IR extract on the A_preb_ of probiotic strains was examined. A 2% overnight monoculture of the selected probiotic strains (*Lactiplantibacillus plantarum* MSD74, *Lacticaseibacillus rhamnosus* ML57, and *Lacticaseibacillus paracasei* MSD108) as well as *Escherichia coli* K12 (K12) was used to inoculate MRS without glucose for *L. plantarum*,* L. rhamnosus*, and *L. paracasei*, and nutrient broth (NB) for K12. Each culture medium contained 2% of various types of postbiotics enriched with either compounds (1–7) or methanol extract. The inoculated media were incubated at 37 °C for 24 h. Serial dilutions of the resulting bacterial growth were then prepared, and after another 24 h of incubation at 37 °C, the populations of *L. plantarum*,* L. rhamnosus*, and *L. paracasei* on MRS agar and K12 on tryptone soya agar (TSA) were counted using the pour plate method^12^. MRS broths containing 2% of each postbiotic without added chemical compounds, as well as broths with 2% glucose and 2% inulin, were used as the negative control, positive control, and prebiotic standard, respectively. A_preb_was calculated following the equation previously described by Dawood, et al^29^.

3 $$\rm{A_{preb}=\frac{log(Lb)_{24}-log(Lb)_{0}PC}{log(Lb)_{24}-log(Lb)_{0}Glucose}- \frac{log(K12)_{24}-log(K12)_{0}PC}{log(K12)_{24}-log(K12)_{0}Glucose}}$$

The prebiotic activity score is denoted as A_preb_, while Log Lb refers to the log CFU/mL of the selected probiotic strains after 24 h (Lb-24) and at the start (Lb-0) of incubation at 37 °C. Log K12 indicates the log CFU/mL of K12 after 24 h (K12-24) and at the start (K12-0) of culture on glucose and the isolated bioactive substances (PC).

### The impact of postbiotics on the enzymatic activity of *Lactobacillus* strains

The enzymatic profiles of *Lactobacillus* strains were assessed following the method outlined by Śliżewska and Chlebicz-Wójcik^30^.

### Statistical analysis

The mean percentages for various parameters, including DPPH and ABTS radical scavenging activities, FRAP, anti-AGEs activity, fructosamine levels, lysine content, free thiol group, protein oxidation, protein aggregation, AGEs-RAGEs interaction, and prebiotic activity scores, were calculated from three independent replicates. SAS 2000 software was used to do a one-way analysis of variance (ANOVA) on these data. When significant main effects were observed, Duncan’s Multiple Range Test was applied for pairwise mean comparisons. Additionally, principal component analysis (PCA) was employed for unsupervised clustering to identify trends and detect outliers within the dataset.

## Results

### Isolation and identification of compounds

Chemical investigation of the methanolic extract of *I. albicans* rhizome led to the isolation and characterization of seven isoflavonoids. They were identified as: irisflorentin (**1**)^31^, 5, 3`,4`-trimethoxy-6,7-methylenedioxyisoflavone (**2**)^32^, irisolidone (**3**) [23], irilone (**4**)^33^, irigenin (**5**)^34^, irisolone methyl ether (**6**)^35^ and irisolone (**7**)^36^. Their structures were elucidated using 1D and 2D NMR and MS analysis.

**Fig. 1:**
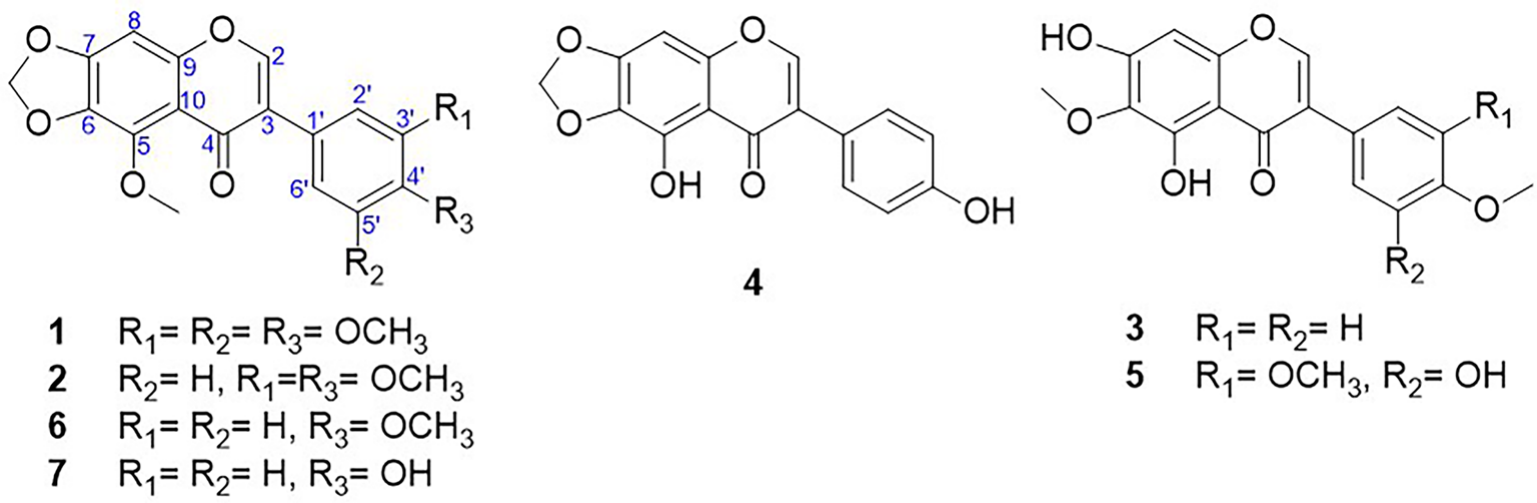
Structures of the isolated compounds from *Iris albicans*.

**Irisflorentin (1)** white needles. ^1^H NMR (600 MHz, DMSO-*d*_6_) δ 8.31 (*s*, 1H, H-2), 7.03 (*s*, 1H, H-8), 6.85 (*s*, 2 H, H-2`, 6`), 6.19 (*s*, 2 H, OCH_2_O), 3.92 (*s*, 3 H, OCH_3_/C-5), 3.81 (*s*, 6 H, OCH_3_/C-3`, 5`), 3.70 (s, 3 H, OCH_3_/C-4`). ^13^C NMR (150 MHz, DMSO-*d*_6_) δ 174.2 (C-4), 154.4 (C-7), 153.2 (C-5), 153.1 (C-3`, 5`), 152.7 (C-2), 141.0 (C-9), 137.8 (C-6), 136.4 (C-4’), 128.0 (C-1`), 124.6 (C-3), 113.7 (C-10), 106.5 (C-2`, 6`), 103.2 (OCH_2_O), 95.8 (C-8), 61.3 (OCH_3_/C-5), 60.5 (OCH_3_/C-3`, 5`), 56.5 (OCH_3_/C-4`).

**5**,**3`**,**4`-trimethoxy-6**,**7-methylenedioxyisoflavone (2).** Orange needles. ^1^H NMR (600 MHz, CDCl_3_) δ 8.47 (*s*, 1H, H-2), 7.16 (*d*, *J* = 2.0 Hz, 1H, H-6`), 7.01 (*dd*,* J* = 8.1, 2.0 Hz, 1H, H-2`), 6.84 (*d*, *J* = 8.1 Hz, 1H, H-5`), 6.76 (*s*, 1H, H-8), 6.19 (*s*, 2 H, OCH_2_O), 3.86 (*s*, 3 H, OCH_3_), 3.80 (*s*, 6 H, 2OCH_3_). ^13^C NMR (CDCl_3_, 150 MHz) δ 181.4 (C-4), 154.1 (C-9); 152.8 (C-7), 150.4 (C-2), 149.1 (C-4`), 146.2 (C-3`), 142.7 (C-5), 130.5 (C-6), 130.4 (C-3), 124.7 (C-1`), 121.8 (C-6`), 115.6 (C-10), 114.6 (C-2`), 111.9 (C-5`), 102.7 (OCH_2_O), 89.3 (C-8), 61.2 (OCH_3_−5), 56.1 (OCH_3_- 3`), 56.0 (OCH_3_−4`).

#### Irisolidone (3).

Yellow amorphous powder. ^1^H NMR (600 MHz, CDCl_3_) δ 13.16 (*s*, 1H, OH/C-5), 7.91 (*s*, 1H, H-2), 7.49 (*d*, *J* = 9.6 Hz, 2 H, H-2`, 6`), 7.02 (*d*, *J* = 9.6 Hz, 2 H, H-3`, 5`), 6.56 (s, 1H, H-8), 4.08 (s, 3 H, OCH_3_/C-4`), 3.89 (*s*, 3 H, OCH_3_/C-6).^13^C NMR (150 MHz, CDCl_3_) δ 181.4 (C-4), 159.9 (C-4`), 155.2 (C-7), 153.5 (C-2), 152.8 (C-5), 152.7 (C-9), 130.4 (C-6), 130.2 (C-2`, 6`), 123.1 (C-1`), 123.0 (C-3), 114.2 (C-3`, 5`), 106.5 (C-10), 93.2 (C-8), 60.9 (OCH_3_/C-6), 55.4 (OCH_3_/C-4`).

**Irilone (4).** Yellowish white needles. ^1^H NMR (600 MHz, CDCl_3_) δ 12.75 (*s*, 1H, OH/C-5), 7.92 (*s*, 1H, H-2), 7.47 (*d*, *J* = 8.4 Hz, 2 H, H-2`, 6`), 6.95 (*d*, *J* = 8.4 Hz, 2 H, H-3`, 5`), 6.54 (*s*, 1H, H-8), 6.13 (*s*, 2 H, OCH_2_O). ^13^C NMR (150 MHz, CDCl_3_) δ 181.3 (C-4), 155.94 (C-4`), 154.2 (C-7), 153.6 (C-2), 152.8 (C-5, 9), 142.7 (C-6), 130.4 (C-2`, 6`), 123.4 (C-1`), 123.0 (C-3), 115.6 (C-3`, 5`), 108.4 (C-10), 102.7 (OCH_2_O), 89.3 (C-8).

**Irigenin (5).** White powder. ^1^H NMR (600 MHz, CDCl_3_) δ 13.13 (*s*, 1H, OH/C-5), 7.92 (*s*, 1H, H-2), 6.76 (*d*, *J* = 2.0 Hz, 1H, H-2’), 6.74 (*d*, *J* = 2.0 Hz, 1H, H-6`), 6.57 (*s*, 1H, H-8), 4.08 (*s*, 3 H, OCH_3_/C-3`), 3.98 (*s*, 3 H, OCH_3_/C-4`), 3.95 (*s*, 3 H, OCH_3_/C-6). ^13^C NMR (150 MHz, CDCl_3_) δ 181.1 (C-4), 155.3 (C-7), 153.4 (C-3`), 153.3 (C-2), 152.7 (C-5), 152.3 (C-9), 149.4 (C-5`), 135.8 (C-4`), 130.5 (C-6), 126.6 (C-1`), 123.2 (C-3), 108.7 (C-6`), 106.5 (C-10), 105.5 (C-2`), 93.2 (C-8), 60.9 (OCH_3_/C-6, 4`), 56.0 (OCH_3_/C-3`).

**Irisolone methyl ether (6).** White powder. ^1^H NMR (600 MHz, DMSO-*d*_6_) δ 8.19 (*s*, 1H, H-2), 7.34 (*d*, *J* = 8.4 Hz, 2 H, H-2`, 6`), 7.0 (*s*, 1H, H-8), 6.80 (*d*, *J* = 8.4 Hz, 2 H, H-3`, 5`), 6.18 (*s*, 2 H, OCH_2_O), 3.90 (*s*, 3 H, OCH_3_/C-5), 3.80 (*s*, 3 H, OCH_3_/C-4`). ^13^C NMR (150 MHz, DMSO-*d*_6_) δ 174.5 (C-4), 157.6 (C-4`), 154.4 (C-7), 153.0 (C-5), 151.5 (C-2), 141.0 (C-9), 136.5 (C-6), 130.7 (C-2`, 6`), 124.7 (C-1`), 122.9 (C-3), 115.3 (C-3`, 5`), 113.8 (C-10), 103.1 (OCH_2_O), 94.1 (C-8), 61.3 (OCH_3_/C-5), 56.2 (OCH_3_/C-4`).

**Irisolone (7). **Colorless plates. ^1^H NMR (600 MHz, DMSO-*d*_6_) δ 8.19 (*s*, 1H, H-2), 7.33 (*d*, *J* = 8.4 Hz, 2 H, H-2`, 6`), 6.99 (*s*, 1H, H-8), 6.80 (*d*, *J* = 8.4 Hz, 2 H, H-3`, 5`), 6.18 (*s*, 2 H, OCH_2_O), 3.90 (*s*, 3 H, OCH_3_/C-5). ^13^C NMR (150 MHz, DMSO-*d*_*6*_) δ 174.5 (C-4), 157.6 (C-4`), 154.4 (C-7), 153.0 (C-5), 151.5 (C-2), 140.9 (C-9), 136.5 (C-6), 130.7(C-2`, 6`), 124.7 (C-1`), 122.9 (C-3), 115.3 (C-3`, 5`), 113.8 (C-10), 103.1 (OCH_2_O), 94.1 (C-8), 61.3 (OCH_3_/C-5).

### Antioxidant properties (DPPH, ABTS, and FRAB) of postbiotics

**Fig. 2:**
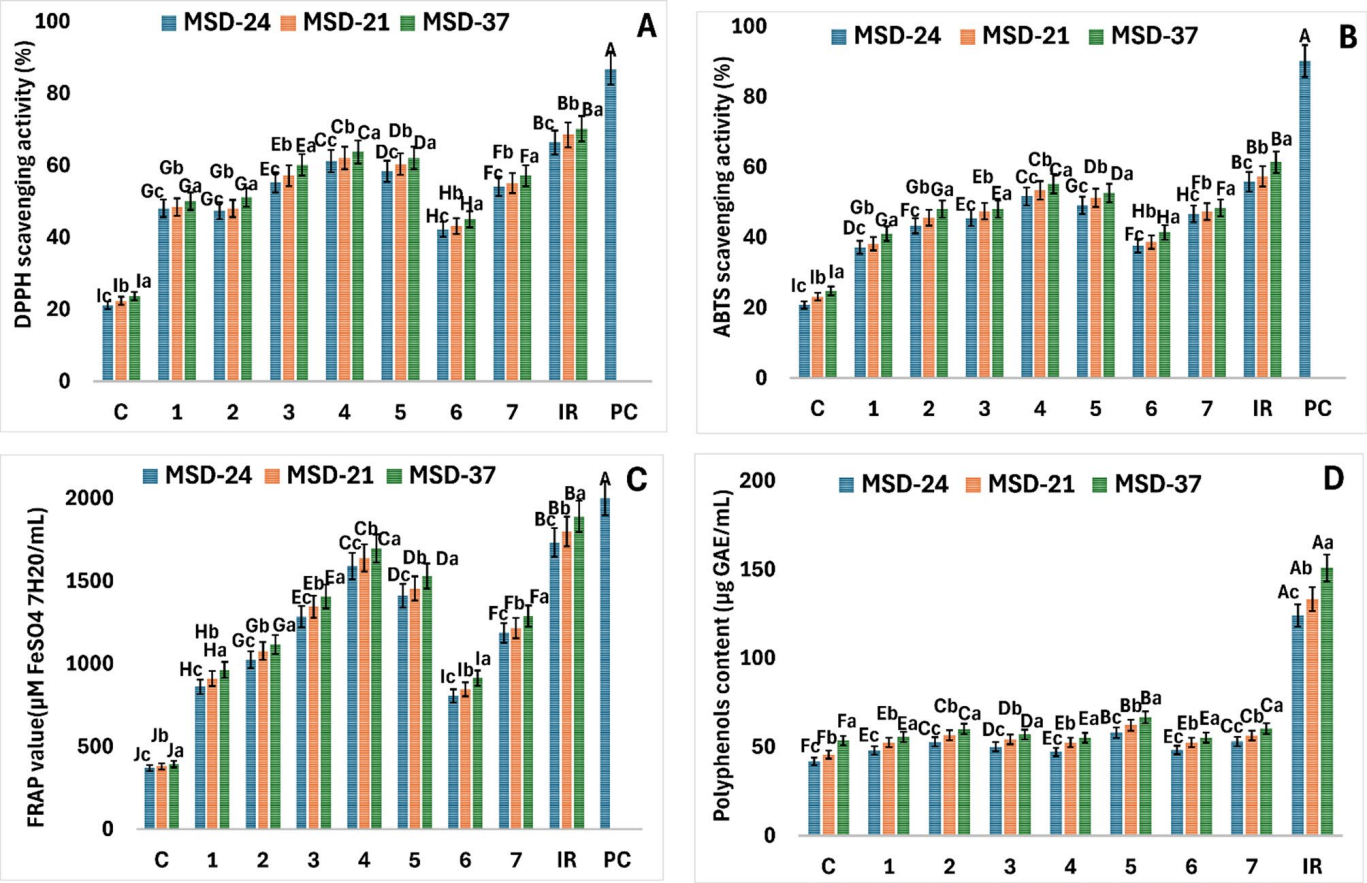
Antioxidant activities, assessed using DPPH (A), ABTS (B), and FRAP (C) assays, together with total phenolic content (TPC) (D), were evaluated for MSD-21, MSD-24, and MSD-37 postbiotics produced by their respective probiotic strains using IR extract or seven isolated isoflavonoid compounds as carbon sources: irisflorentin (**1**), 5,3`,4`-trimethoxy-6,7-methylenedioxyisoflavone (**2**), irisolidone (**3**), irilone (**4**), irigenin (**5**), irisolone methyl ether (**6**), irisolone (**7**) and glucose as a control. Ascorbic acid was used as a positive control (PC). Data are presented as mean ± SD (*n* = 3). Different capital letters indicate statistically significant differences among individual compounds, while lowercase letters denote significant differences among postbiotic types (*p* < 0.05).

Overall, both the type of carbon source and the bacterial strain significantly influenced the antioxidant properties of postbiotics. However, strain-dependent differences were more pronounced, with MSD37 consistently outperforming MSD21 and MSD24 across all carbon sources, while IR extract generally enhanced antioxidant activity compared with individual compounds and glucose. Figure 2 (A–C) illustrates the variations in DPPH radical scavenging activity, ABTS, and FRAP among postbiotics produced using different pure compounds (**1**–**7**) or IR extract as carbon sources. All treated samples exhibited a higher percentage of scavenging activity (DPPH, ABTS, and FRAP) compared to their corresponding controls. Postbiotics derived from MSD37 exhibited higher scavenging activities (DPPH, ABTS, and FRAP) than those from MSD-21 and MSD-24 when produced using compounds (**1**–**7**), IR extract, or glucose (control) as carbon sources (Fig. 2A–C). Postbiotics produced using compounds (**1**–**7**) or IR extract as carbon sources exhibited DPPH, ABTS, and FRAP activities that were approximately 2.0–3.0, 1.6–2.5, and 2.2–4.8 times higher, respectively, than those produced using glucose (control). The increase in antioxidant activity (DPPH, ABTS, and FRAP) was significantly higher (*p* < 0.05) in postbiotics produced using IR-extract compared to those produced using individual compounds (Fig. 1A-C). The greatest antioxidant activity was observed with MSD-37 postbiotic produced using IR extract as a carbon source, followed by those produced using **4**, **5**, **3**, and **7**. Conversely, MSD-24 postbiotic derived from compounds **6**, **1**, and **2** as carbon sources showed significantly lower antioxidant activity (*p* < 0.05). Overall, all postbiotic treatments exhibited lower antioxidant activities compared to the positive control, ascorbic acid, across the DPPH, ABTS, and FRAP assays.

The highest TPC was recorded for MSD-37 postbiotic paired with IR extract, **5**, **2**, and **7** (151, 66.8, 60.2, and 60.34 µg GAE/mL, respectively). In contrast, the TPC of MSD-24 postbiotic was significantly lower (*p* < 0.05) when combined with compounds **1** and **5**, recording values of 47.3 and 48 µg GAE/mL, respectively (Fig. 2D).

### Antiglycation of postbiotics enriched with isoflavonoids or IR extract

**Fig. 3:**
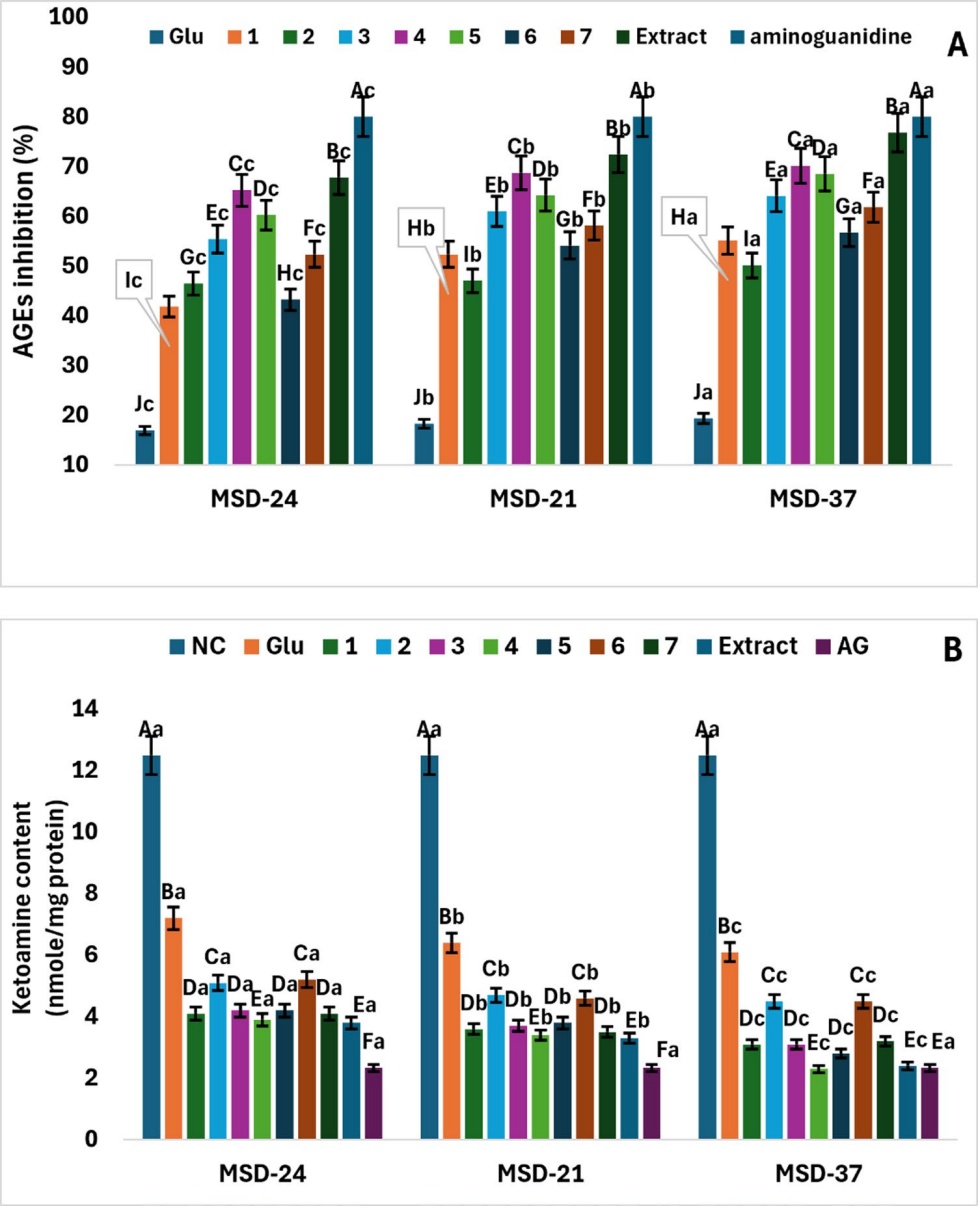
The inhibitory effects of postbiotics (MSD-24, MSD-21, and MSD-37) on advanced glycation end-products (AGEs) formation (A) and formation of ketoamine in the early stage of protein glycation (B). Numbers **1**–**7** represent the isoflavonoid compounds utilized (irisflorentin (**1**), 5,3`,4`-trimethoxy-6,7-methylenedioxyisoflavone (**2**), irisolidone (**3**), irilone (**4**), irigenin (**5**), irisolone methyl ether (**6**), and irisolone (**7**), Glu denotes the postbiotics that employed glucose as the carbon source, NC indicates the negative control (without postbiotics), and Aminoguanidine (AG) is used as the positive control. The data are presented as the mean ± SD (*n* = 3). Different capital letters signify statistically significant differences between the individual compounds (*p* < 0.05), while lowercase letters indicate significant differences among the various types of postbiotics.

Overall, both the carbon source and the producing strain influenced the antiglycation activity of the postbiotics; however, strain-dependent differences were more pronounced, with MSD37 consistently showing stronger inhibition of AGE formation than MSD21 and MSD24 across most carbon sources, while specific isoflavonoids (especially IR extract, irilone, irigenin, and irisolidone) further enhanced this effect. Advanced Glycation End-Products (AGEs) are signaling proteins linked to various vascular and neurological complications in individuals with diabetes. In this study, the antiglycation assay was conducted to evaluate the effectiveness of postbiotics (MSD-21, MSD-24, and MSD-37), which were produced using various pure compounds derived from IR or its extract as carbon sources, in inhibiting the formation of advanced glycation end products (AGEs). In this assay, fructose functioned as a glycating agent, and bovine serum albumin was used as the model protein. All the tested postbiotics derived from pure chemical compounds (**1**–**7**) or IR extract inhibited AGE formation in a manner dependent on the specific type of compound or extract, but their effectiveness was still less than that of the positive control (AG) (Fig. 3-A). The postbiotics of MSD-37 show a significantly greater inhibitory effect against AGEs (*p* < 0.05) compared to both MSD-21 and MSD-24, regardless of the type of isoflavonoid compounds or IR extract used as a carbon source in the MRS environment. It was also observed that using IR extract irilone (**4**) as a carbon source led to a significantly higher increase in antiglycation activity across all postbiotic types (*p* < 0.05), followed by irilone (**4**) and irigenin (**5**). In the presence of MSD-37 postbiotic, these compounds inhibited 70.1, 68.5, and 64.3% of AGEs, respectively (Fig. 3A). In contrast, 5,3,4-trimethoxy-6,7-methylenedioxyisoflavone (**2**) showed the lowest antiglycation activity when used as a carbon source for MSD‑21 and MSD‑37 postbiotics (approximately 50% inhibition, as indicated by the corresponding significance letters in Fig. 3A), whereas, for MSD‑24, irisflorentin (**1**) exhibited an even lower activity (around 40% inhibition, as indicated by its significance letter in Fig. 3A).

#### Early stage

##### Influence of postbiotics on fructosamine content

Variation in early antiglycation responses was driven by both the producing strain and the nature of the carbon source. Among these factors, MSD37 exerted the strongest overall suppression of fructosamine formation, while IR extract and irilone (**4**) consistently yielded the most potent substrate-dependent enhancements. Ketoamine, one of the Amadori products, was identified and is illustrated in Fig. 3-B to represent the early stages of protein glycation. The ketoamine concentration in the glycated BSA (without postbiotics) was measured at 12.50 nmol/mg. The addition of various types of postbiotics (MSD-21, MSD-24, and MSD-37) resulted in a significant reduction (*p* ≤ 0.05) in ketoamine content compared to glycated BSA. It was also observed that the use of MSD-37 recorded the highest reduction in ketoamine formation compared to other types of postbiotics (MSD-21 and MSD-24) (Fig. 3-B). The use of IR extract as a carbon source produced postbiotics with strong inhibitory activity against ketoamine formation in glycated BSA. A similar effect was observed with irilone (**4**), with no significant difference between IR extract and irilone (*p* > 0.05) (Fig. 3-B). Moreover, the inhibitory activity of MSD-37 postbiotic did not differ significantly from that of AG (positive control) when IR extract or irilone (**4**) was used as the carbon source (*p* > 0.05). Among the tested substrates, irilone (**4**) yielded the highest inhibitory activity, followed by irigenin (**5**), irisolidone (**3**), and irisflorentin (**1**), which showed comparable inhibitory effects at the same level of significance (*p* > 0.05) (Fig. 3-B).

##### Effect of postbiotics on free lysine content

**Fig. 4:**
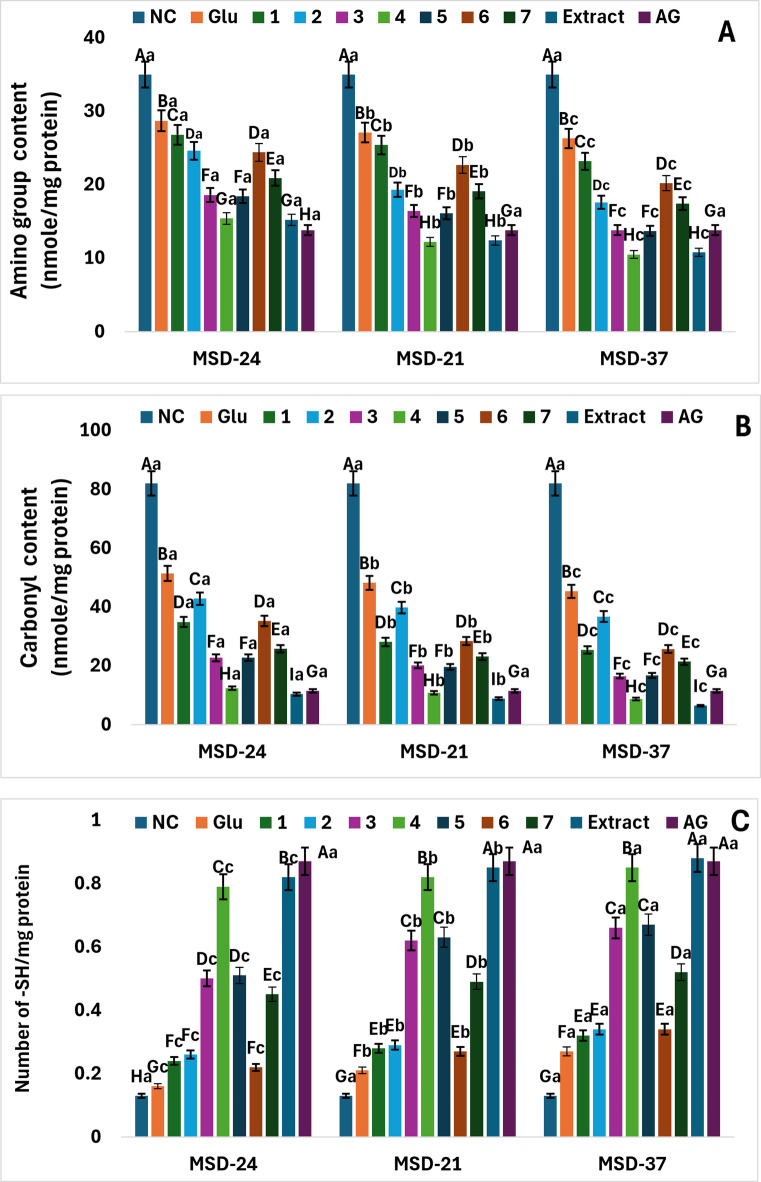
The impact of postbiotics (MSD-24, MSD-21, and MSD-37) on the free lysine content (A), carbonyl group content of products (B), and thiol group levels (C) at the final stage of protein glycation. Numbers **1**–**7** represent the isoflavonoid compounds utilized (irisflorentin (**1**), 5,3`,4`-trimethoxy-6,7-methylenedioxyisoflavone (**2**), irisolidone (**3**), irilone (**4**), irigenin (**5**), irisolone methyl ether (**6**), and irisolone (**7**), Glu denotes the postbiotics that employed glucose as the carbon source, NC indicates the negative control (without postbiotics), and Aminoguanidine (AG) is used as the positive control. The data are presented as the mean ± SD (*n* = 3). Different capital letters signify statistically significant differences between the individual compounds (*p* < 0.05), while lowercase letters indicate significant differences among the various types of postbiotics.

Dependence of lysine protection involved both the producing strain and the type of carbon source, with MSD37 generally conferring the strongest preservation of free lysine residues, and IR extract and irilone (**4**) acting as the most effective substrates across strains. Figure 4-A illustrates the number of lysine residues modified in BSA after the addition of different postbiotics following a 7-day incubation period. The lysine residues modified by MSD-37 postbiotics were fewer compared to those modified by other postbiotic types (MSD-21 and MSD-24). Supplementation with IR extract improved the effectiveness of various postbiotics in reducing the number of lysine residues in glycated BSA, with irilone (**4**) showing the next highest impact, followed by irigenin (**5**) and irisolidone (**3**), which exhibited equal effectiveness.

#### Oxidation stage

##### Impact of postbiotics on protein carbonyl content

Both the producing strain and the carbon source influenced protein carbonyl suppression, with MSD37 generally exerting the strongest protective effect, and I. albicans extract providing the most effective substrate, followed closely by irilone (**4**). To evaluate protein oxidation induced by the glycation process, protein carbonyl levels were monitored over 7 days, as illustrated in Fig. 4-B. The carbonyl content in glycated protein showed a significant increase; however, treatment with postbiotics (MSD-21, MSD-24, and MSD-37) effectively inhibited this rise. After 7 days, the MSD-37 postbiotic, utilizing isoflavonoid compounds, achieved a reduction in carbonyl content ranging from 55.24 to 92.19%, depending on the type of isoflavonoid used.

##### Influence of postbiotics on the thiol group in glycated BSA

Both the bacterial strain and the carbon source influenced the preservation of thiol groups, with MSD37 consistently offering the strongest protection, particularly when grown on IR extract and specific isoflavonoids. The free thiol groups were quantified using Ellman’s assay, and the results are shown in Fig. 4-C. The findings demonstrated a substantial increase in free thiol groups in glycated BSA following treatment with all types of postbiotics. Compared to glycated BSA (without postbiotics as a negative control), this increase was pronounced in glycated BSA incubated with MSD-37 postbiotics, which used IR methanolic extract as a carbon source for postbiotic production, followed by irilone (**4**), then irigenin (**5**), and irisolidone (**3**).

#### Cross-linking stage

##### Impact of postbiotics on protein aggregation

**Fig. 5:**
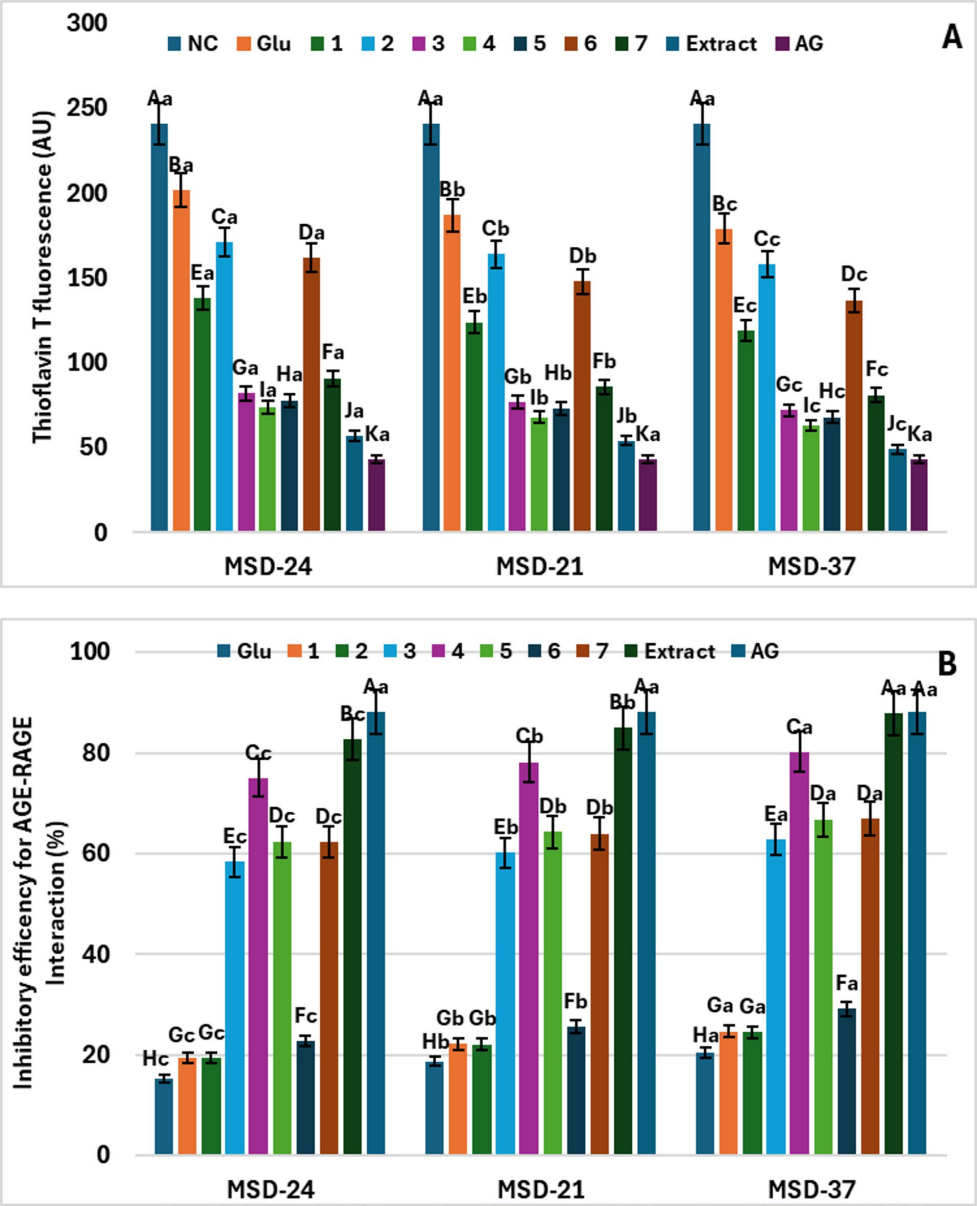
The impact of postbiotics (MSD-24, MSD-21, and MSD-37) on protein aggregation levels, assessed through thioflavin T fluorescence in glycated BSA (A), and their effect on the binding of RAGE with glycated proteins (B). Numbers **1**–**7** represent the isoflavonoids utilized (irisflorentin (**1**), 5,3`,4`-trimethoxy-6,7-methylenedioxyisoflavone (**2**), irisolidone (**3**), irilone (**4**), irigenin (**5**), irisolone methyl ether (**6**), and irisolone (**7**), Glu denotes the postbiotics that employed glucose as the carbon source, NC indicates the negative control (without postbiotics), and Aminoguanidine (AG) is used as the positive control. The data are presented as the mean ± SD (*n* = 3). Different capital letters signify statistically significant differences between the individual compounds (*p* < 0.05), while lowercase letters indicate significant differences among the various types of postbiotics.

Bacterial strain background and carbon sources jointly influenced the inhibition of amyloid aggregation, with MSD37 consistently showing the strongest effect, especially when grown on I. albicans extract and certain isoflavonoids. The glycation of albumin leads to the formation of amyloid fibrils characterized by a cross-beta structure. The amyloid content in glycated albumin samples was quantified using the binding property of amyloid fibrils to the dye thioflavin T. As shown in Fig. 5A, the MSD-37 postbiotic produced using IR extract as a carbon source demonstrated a notable inhibition of amyloid aggregation (97.68% at a concentration of 2 mg/mL after 7 days), followed by MSD-37 postbiotics derived from irilone (**4**) and irigenin (**5**). This highlights their ability to prevent conformational changes in albumin during glycation. However, all types of postbiotics with different isoflavonoid compounds exhibited lower inhibitory effects on amyloid formation compared to Aminoguanidine, the standard inhibitor of beta-amyloid formation.

#### Signaling stage

##### The influence of postbiotics on AGE-RAGE interaction

Strain identity and carbon source jointly shaped the inhibition of AGE–RAGE interaction, with MSD37 generally exerting the strongest effect, particularly when combined with I. albicans extract and selected isoflavonoids. In this context, Fig. 5B illustrates the inhibitory efficiency of the AGE-RAGE interaction. All postbiotics (MSD-21, MSD-24, and MSD-37), whether produced using IR extract, individual isoflavonoid compounds, or glucose (control) as carbon sources, exhibited inhibitory effects on AGE-RAGE binding. Among them, MSD-37 showed the highest inhibitory rate. The use of IR extract as a carbon source yielded the greatest inhibitory efficiency across all postbiotics, followed by irilone (**4**) and subsequently irigenin (**5**). However, the inhibitory effects of all treatments remained lower than those of aminoguanidine. These findings suggest that postbiotics not only mitigate glycation by reducing the formation of glycation derivatives, glycated residues, and Amadori products but also prevent AGE-RAGE binding, thereby inhibiting downstream aging-related cellular pathways (Fig. 5B).

### Prebiotic activity score (A_preb_) of postbiotics

**Fig. 6:**
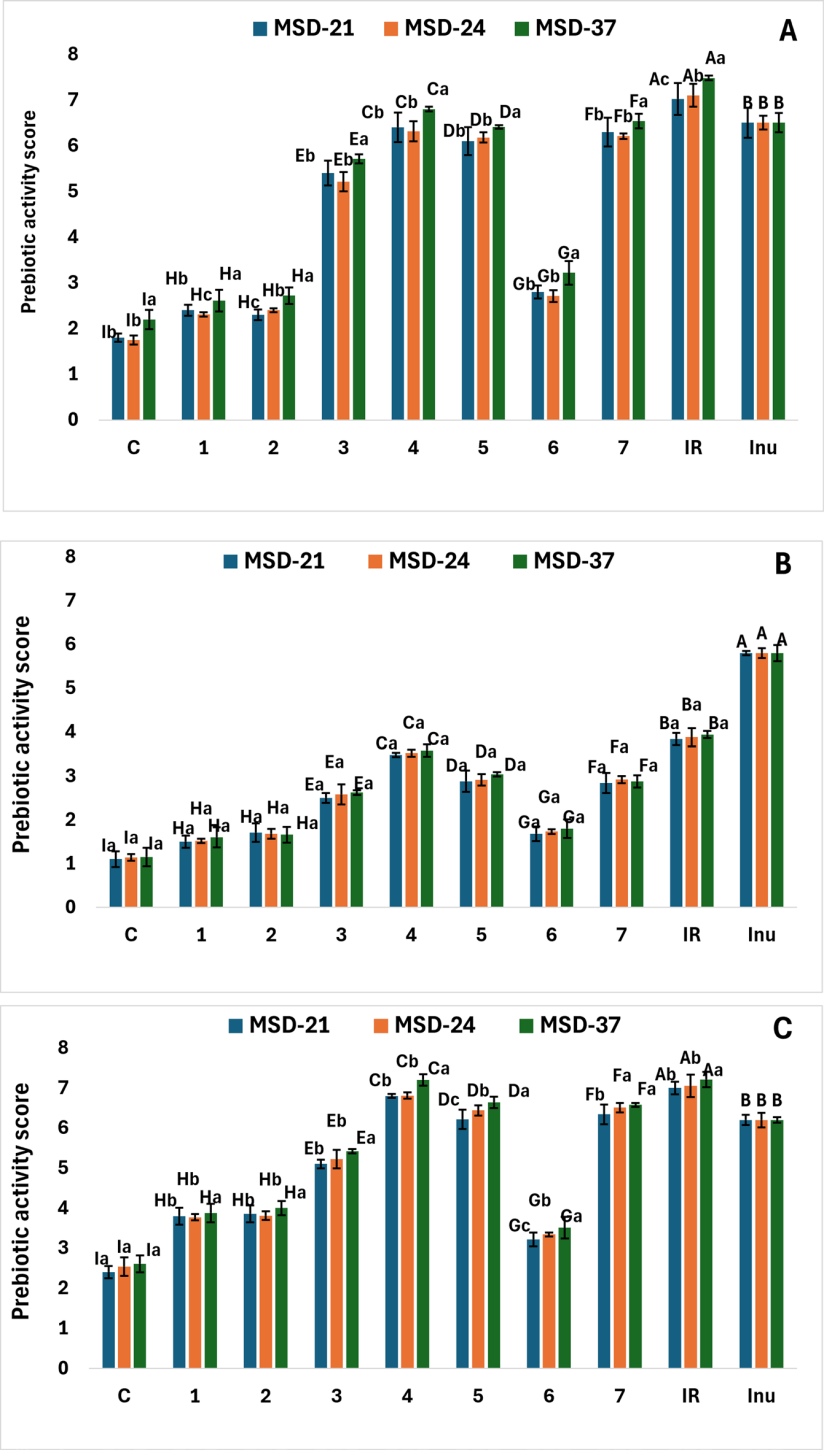
Prebiotic activity scores (A_preb_) for *L. plantarum* (A), *L. rhamnosus* (B), and *L. paracasei* (C) were measured when paired with three different types of postbiotics (MSD-21, MSD-24, and MSD-37) produced by their respective probiotic strains using IR extract or seven isolated isoflavonoid compounds as carbon sources: irisflorentin (**1**), 5,3`,4`-trimethoxy-6,7-methylenedioxyisoflavone (**2**), irisolidone (**3**), irilone (**4**), irigenin (**5**), irisolone methyl ether (**6**), and irisolone (**7**). Control (C) denotes the postbiotics that employ glucose as the carbon source, and inulin is used as a prebiotic standard. Capital letters indicate statistically significant differences among the individual compounds (*p* < 0.05), while lowercase letters indicate statistically significant differences among the different postbiotic types. Vertical bars represent the standard deviations of the means for the treatments.

Both the producer strain and the carbon source influenced prebiotic activity, with MSD37‑derived postbiotics and IR extract generally giving the highest A_preb_ values, particularly for *L. plantarum* MSD74 and *L. paracasei* MSD108.The Prebiotic activity scores shown in Fig. 6 were calculated based on the growth patterns of the probiotic strains using Eq. (3). The highest A_preb_ values were found for *L. plantarum* MSD74 and *L. paracasei* MSD108 when incubated with all postbiotics produced using IR extract as the carbon source, compared to other treatments. However, the highest A_preb_ value for *L. rhamnosus* ML57 was observed when incubated with inulin (a standard prebiotic) compared to other treatments (Fig. 6). The A_preb_ values for *L. paracasei* MSD108 incubated with postbiotic MSD-37 produced using compounds **4**, **5**, and **7** as carbon sources were 7.2 ± 0.05, 6.64 ± 0.14, and 6.58 ± 0.21, respectively. *L. plantarum* MSD74 grown in postbiotic MSD-37 derived from compounds **4**, **7**, and **5** as the carbon sources showed slightly lower of A_preb_ values of 6.8 ± 0.04, 6.54 ± 0.05, and 6.41 ± 0.16, respectively (Fig. 6). In contrast, the lowest A_preb_ values (*p* < 0.01) were recorded for *L. rhamnosus* grown in postbiotic MSD-21 produced using compounds **1**, **6**, and **2**, with values of 1.5 ± 0.14, 1.68 ± 0.21, and 1.71 ± 0.17, respectively.

### The Impact of postbiotics on the enzymatic activities of probiotic strains

**Fig. 7:**
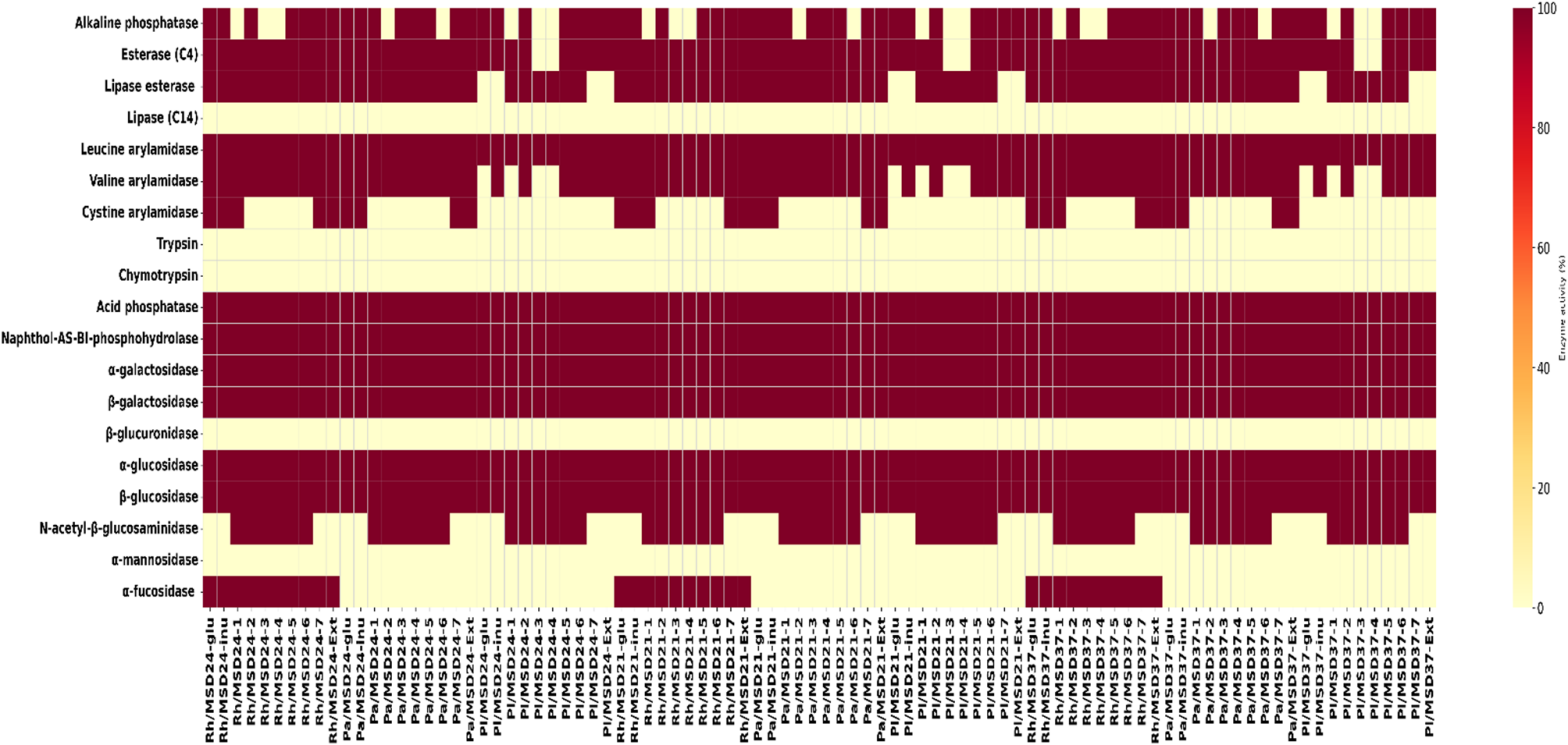
The heatmap illustrates the enzymatic profiles of the selected probiotic strains (*L. paracasei* (Pa), *L. plantarum* (Pl), and *L. rhamnosus* (Rh)) in response to postbiotics (MSD-24, MSD-21 and MSD-37) produced by their respective probiotic strains using IR extract or seven isolated isoflavonoid compounds as carbon sources: irisflorentin (**1**), 5,3`,4`-trimethoxy-6,7-methylenedioxyisoflavone (**2**), irisolidone (**3**), irilone (**4**), irigenin (**5**), irisolone methyl ether (**6**), and irisolone (**7**).“Inu” represents inulin as a prebiotic standard, while “Glu” serves as the positive control using glucose. Red indicates a positive enzymatic reaction, whereas off-white signifies the absence of enzyme activity.

Based on the API^®^ ZYM assay, all probiotic strains showed consistent activities of leucine arylamidase, acid phosphatase, naphthol-AS-BI-phosphohydrolase, β-galactosidase, and α/β-glucosidase, regardless of the postbiotic treatment. In contrast, no lipase, trypsin, chymotrypsin, β-glucuronidase, α-mannosidase, or α-fucosidase activities were detected for any strain under the tested conditions, except for *L. rhamnosus* (Fig. 7). Carbon source–dependent differences were observed for cystine arylamidase, alkaline phosphatase, and N-acetyl-β-glucosaminidase. Postbiotics produced with compound **7** or IR extract activated cystine arylamidase in *L. rhamnosus*, while in *L. paracasei* this enzyme was induced by postbiotics containing compounds **1** or **7** or the IR extract. Postbiotics generated with isoflavonoids **2** and **5**–**7**, as well as IR extract, stimulated alkaline phosphatase activity in *L. rhamnosus* and *L. plantarum*, whereas activation in *L. paracasei* was associated with postbiotics derived from compounds **1**, **3**, **4**, **5**, **7**, or the extract. In contrast, these postbiotics, including those based on compound **7** and IR extract, tended to suppress N-acetyl-β-glucosaminidase activity (Fig. 7).

### PCA of the Multifunctional Activity of Postbiotics

**Fig. 8:**
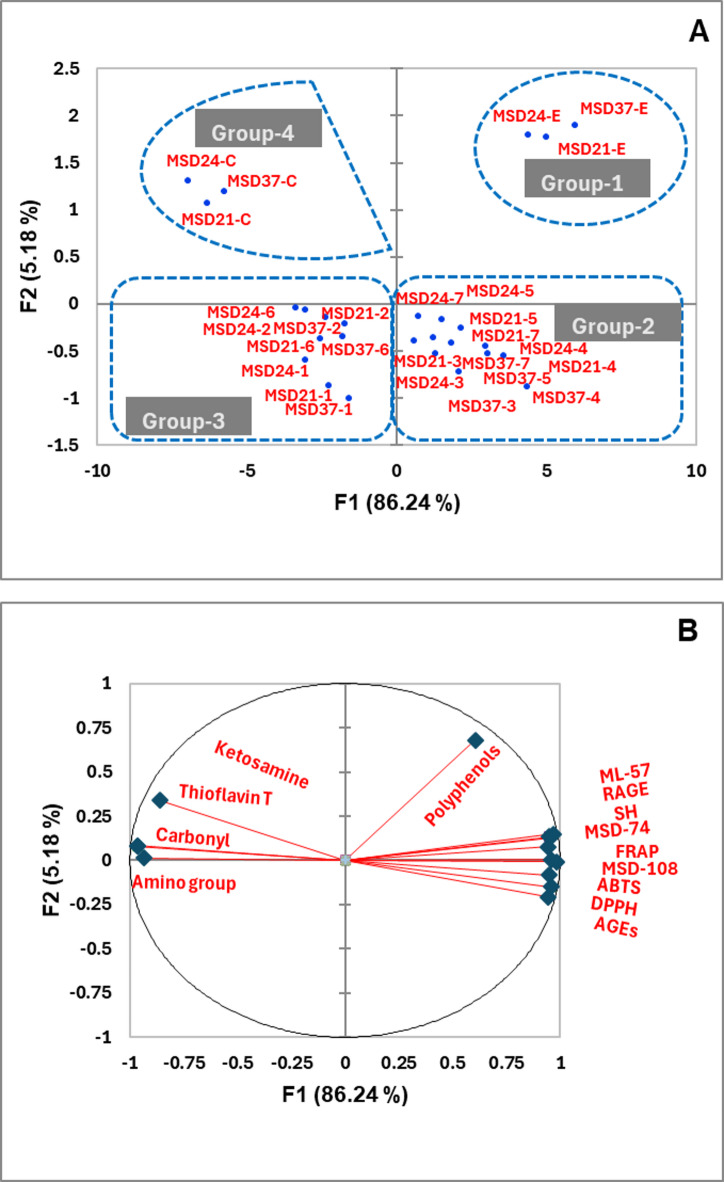
(**A**) Biplot of the principal component analysis (PCA) of the impact of the postbiotics enriched assays with *Iris albicans* extract and isolated isoflavonoids on the antioxidant activity (DPPH, ABTS, and FRAP), total phenolic compounds, Antiglycation activity, and prebiotic activity score (A_preb_). (**B**) Analysis of the correlations among variables.

The principal component analysis (PCA) of the antioxidant, antiglycation, and prebiotic activities of the postbiotics (MSD‑21, MSD‑24, and MSD‑37) using two principal components (PCs) accounted for 91.42% of the total variability (Fig. 8A). PC1, which explained 86.24% of the variance, encompassed all evaluated parameters except polyphenol content, whereas PC2, accounting for a further 5.18% of the variance, was mainly associated with polyphenol content (Fig. 8A). PCA separated the samples into four groups: groups 1 and 2 were located on the positive side of PC1, while groups 3 and 4 were positioned on the negative side. Group 1 comprised all postbiotics (MSD‑21, MSD‑24, and MSD‑37) produced using IR extract as a carbon source and was characterized by the highest antioxidant activity (DPPH, ABTS, FRAP), potent antiglycation activity, elevated SH group content, superior prebiotic activity across probiotic strains, and reduced protein oxidation (carbonyls), lysine residue modification, protein aggregation, and AGEs–RAGE interaction. Group 2 followed group 1 for the same postbiotic types across most parameters but was distinguished by a lower polyphenol content. In contrast, groups 3 and 4 exhibited generally lower values for these protective parameters, placing them in the negative region of PC1; notably, group 4, corresponding to glucose‑derived postbiotics, showed the highest levels of protein oxidation, lysine residue modification, protein aggregation, and AGEs–RAGE interaction compared with group 3 (Fig. 8A).

To further elucidate the underlying structure, a PCA was performed to integrate these antioxidant, antiglycation, and prebiotic activity scores and to identify the key variables responsible for discriminating between postbiotics produced with IR extract (high‑efficiency group) and those produced with glucose (low‑efficiency group). The first principal component (PC1) accounted for 86.24% of the variance, with PC2 explaining an additional 5.18%, confirming that most of the multivariate information is captured along PC1. As shown in the loading table (Table S1), PC1 exhibited strong positive loadings for DPPH, ABTS, FRAP, total polyphenols, AGEs, SH groups, AGE–RAGE readout, and prebiotic activity indices for MSD‑74, ML‑57, and MSD‑108, while ketosamine, protein carbonyls, free amino groups, and Thioflavin T loaded strongly in the opposite direction. The corresponding variable‑contribution table (Table S2) demonstrates that these variables collectively contribute the largest proportions to PC1, underscoring their dominant role in sample discrimination. Consistently, the PCA score plot shows that IR‑extract‑derived postbiotics cluster on the positive side of PC1, whereas glucose‑derived postbiotics are shifted toward the negative side, indicating that the IR extract simultaneously enhances antioxidant capacity and modulates antiglycation/oxidative damage markers and prebiotic activity scores in a coordinated fashion. Together, the group structure in the score plot and the loading and contribution patterns (Tables S1 and S2) support a superior effect of the IR extract, whereby improvements across multiple, correlated biochemical endpoints jointly drive the separation of the high‑efficiency IR group from the low‑efficiency glucose group along PC1 (Fig. 8B).

## Discussion

In this study, replacing glucose as a carbon source in synthetic media (MRS) with isoflavonoid compounds or IR extract for the cultivation of probiotic strains led to the production of postbiotics with high antioxidant capacity, anti-AGEs properties, and enhanced prebiotic activity.

The buildup of reactive nitrogen species (RNS) and reactive oxygen species (ROS) leads to oxidative stress, contributing to aging and various diseases through oxidative damage to lipids, proteins, and nucleic acids^37,38^. Postbiotics have notable antioxidant effects that help reduce oxidative stress, safeguard cells from harm, and promote general health. Numerous bioactive components, including peptides, organic acids, short-chain fatty acids (SCFAs), polysaccharides, vitamins, and phenolic compounds, are responsible for their antioxidant capacity. By scavenging free radicals, activating antioxidant enzymes, chelating metal ions, preventing lipid peroxidation, lowering inflammation, and modifying redox signaling pathways, these substances support the antioxidant effects of postbiotics^39^. Using the methanolic extract of *Iris albicans*as a carbon source produced postbiotics with superior antioxidant capacity compared to those generated using individual pure isoflavonoid compounds, indicating that the antioxidant activity depends on the quality of supplementation. This enhanced effect is likely due to the extract’s rich diversity of phytochemicals, including polyphenols, flavonoids, and other secondary metabolites, which together confer a superior effect of the complex extract compared with isolated compounds^40^. Additionally, the complex matrix of the extract may improve the bioavailability and stability of active constituents, further contributing to its functional potency. These findings somewhat agree with those of Elbermawi, et al^12^., who indicated that myricetin-3-*O*-α-L-rhamnoside outperformed the antioxidant activity of *Guizotia abyssinica* extract, but genkwanin and 1-heneicosanol were inferior to it. Postbiotics’ antioxidant activity is influenced by the isoflavonoids’ composition. For instance, utilizing compound **4** as a carbon source in MRS medium resulted in the maximum antioxidant activity for all postbiotic types, followed by compounds **5**, **3**, and **7**.

Since the antioxidant compounds give one hydrogen atom and one electron to produce stable radicals, the configuration and level of hydroxylation on the B ring were important determinants affecting their capacity to scavenge different types of radicals. Both theoretical and practical research have demonstrated the potent antioxidant qualities of flavonols^41^. The compound with the greatest antioxidant activity in this investigation was irilone (**4**), which was closely followed by irigenin (**5**). Conjugation is made possible by the 2,3-double bond and 4-oxo group, and flavonols’ robust antiradical properties are enhanced by the hydroxyl group’s ability to create hydrogen bonds with hydroxyl groups on the B ring. The hydroxyl group on the B ring’s 4`-carbon is probably what gives irilone (**4**) its greater antioxidant action; this is consistent with earlier research showing that more hydroxyl groups on the B ring boost antioxidant capacity. The B ring of quercetin, a well-known flavonoid with strong antioxidant properties, contains catechol groups^42^. In contrast to the molecule irilone (**4**), the compound irisolidone (**3**) has a lower antioxidant capacity (DPPH, FRAP, and ABTS) due to the substitution of a methoxy group for the hydroxyl group on the 4’ carbon atom of the B ring. In the same way, substituting a hydroxyl group for the fifth carbon of the A ring in irisolone (**7**) decreased its antioxidant capacity in comparison to irilone (**4**). In addition, adding a hydroxyl group to the 4’ carbon atom of the B ring in irisolone (**7**) elevated its antioxidant capacity in comparison to irisolone methyl ether (**6**). *O*-methylation’s decrease in antioxidant activity might be the consequence of steric effects that interfere with molecular planarity^42,43^.

The changes in replacement within the methoxy and hydroxyl groups of a flavonoid do not always determine the scavenging ability of the flavonoid; however, the configurational effect is most sensitive regarding the B-ring. The 6`-OMe/4`-OH configuration allows for promotion of co-planarity in its DPPH scavenging results, as opposed to the 6`-OH/4`-OMe configuration that nullifies the scavenging^44^. Prolonged use of Irigenin improves and augments protective mechanisms, such as SOD, GSH-px, and GSH, which inhibit oxidative stress. It also decreases the formation of ROS induced by doxorubicin (DOX)^45^ and lowers the monocation radical of ABTS in a dose-dependent manner. The compound’s high methoxy and hydroxy group concentration is responsible for its potent antioxidant action^46^. Ibrahim and his colleagues further confirmed irigenin’s strong antioxidant properties in 2017 by showing that it inhibits DPPH^47^.

Advanced Glycation End-products are signaling proteins linked with different complications, like those resulting from the nervous system or the vascular changes induced by diabetes. The formation of these proteins arises from many heterogeneous compounds that can be formed by the non-enzymatic reaction of free amino groups with either aldehydes or sugars (the Maillard reaction). AGE formation occurs during oxidative stress or hyperglycemia, resulting in the conversion of reversible Schiff-base intermediates to covalently bound Amadori products, which subsequently undergo further chemical rearrangements, resulting in the formation of an irreversibly bound AGE^48^. Methods for measurement and detection of AGE have been innovatively upgraded in recent years, and this may perhaps have a positive application shortly for the bedside care of diabetic patients^49^. It is documented that AGEs cause vascular complications of cardiovascular diseases and diabetes; hence, interest in their pharmacological inhibition has increased. This led to numerous clinical and preclinical investigations as well as a flurry of experimental activity^50^. This study investigated the impact of various postbiotics (MSD-21, MSD-24, and MSD-37, produced by *L. fermentum* MSD-21, *L. casei* MSD-24, and *L. reuteri* MSD-37, respectively) combined with or without isoflavonoid compounds from *Iris albicans* or its methanolic extract as a carbon source for probiotic strains. The focus was on assessing multiple parameters related to albumin glycation and protein oxidation mitigation. The findings revealed that all postbiotics effectively inhibited fluorescent AGE formation, with MSD-37 paired with *Iris albicans* extract showing the strongest effect, followed in increasing order by irilone (**4**), irigenin (**5**), risolidone (**3**), and irisolone (**7**). Glycation of BSA by fructose resulted in a significant rise in lysine content, protein carbonyl content, fructosamine levels, and protein aggregation. However, the addition of postbiotics to these systems significantly suppressed these effects.

The findings of the current study align with those of Lin, et al^3^. Despite not using phenolic compounds, flavonoids, or isoflavonoids as carbon sources to produce fermented supernatants, and instead relying on the conventional carbon source glucose. They reported that fermented supernatants of *L. plantarum *GKM3 and GKK2 can effectively inhibit protein glycation by either capturing the glycation agent methylglyoxal or shielding protein functional groups from methylglyoxal-induced reactions. Similarly, Kumar, et al^9^. reported that cell-free extracts of *L. paracasei* MKU2, *L. casei* MKU1, *L. paracasei* MKU7, *L. pentosus* MKU3, *L. delbrueckii* MKU10 and *L. delbrueckii *GERU3 act as potent inhibitors of AGEs formation. Kaga, et al^51^. investigated the anti-glycation properties of a LAB-fermented aqueous extract of the microalga Nostoc commune, which demonstrated a 68% inhibition in the formation of advanced glycation end-products (AGEs).

There is a direct relationship between antioxidant capacity and the inhibition of AGEs formation, fructosamine levels, carbonyl protein content, protein aggregation, and the formation of a covalent bond between the carbonyl group of sugar and the free amino group of proteins. This explains the enhanced effect of postbiotics associated with the *Iris albicans* extract, followed by irilone (**4**) and then irigenin (**5**)^27^. According to Wu, et al^52^., the antioxidant activities displayed by polyphenols to reduce free radical generation may significantly protect against AGE formation. The pronounced anti-aging effect of MSD-37 related to the IR extract could be linked to its higher polyphenolic concentration (151 mg GAE/mL) compared to MSD-21-IR (133.4 mg GAE/mL) and MSD-24-IR (124.2 mg GAE/mL), suggesting that increased polyphenolic content may contribute to its biological activity. Recent research has now demonstrated that polyphenolic compounds available in edible plants are protective against protein glycation induced by monosaccharides^53,54^. Another nucleophilic moiety in BSA that covalently reacts with the carbonyl group of sugars originates from the thiol group of the cysteine residue^28^. All types of postbiotics in this study increased free sulfhydryl levels compared with glycated BSA. This is consistent with Lin, et al^3^., that stated the use of GKM3-and GKK2-fermented supernatants can increase free sulfhydryl content compared to glycated BSA.

The postbiotics evaluated in this study inhibit the interaction between advanced glycation end-products (AGEs) and their receptor (RAGE), thereby preventing the initiation of cellular signaling processes associated with aging. In doing so, they reduce glycation by blocking the formation of Amadori products, methylglyoxal (MG) derivatives, and other glycated residues^3^. Moreover, the multi-ligand receptor for advanced glycation end-products (RAGE) plays a significant role in the etiology and progression of several diseases, including cardiovascular diseases, cancer, diabetic complications, nephropathy, and neuropathy^55^. Hence, AGE-RAGE ligation is the crucial event that makes the difference between the appearance and the genesis of aging-related diseases^56^. Initially, our research showed that all types of postbiotics, MSD-21, MSD-24, and MSD-37, could strongly inhibit the interaction between AGE and RAGE. This could be a part of the mechanisms behind the biological effects of these postbiotics, which have been exposed in another earlier study^57,58^. The interaction between the ligand and RAGE stimulates NF-kappa B (NF-kB) and increases the production of adhesion molecules, cytokines, and oxidative stress^3^. Postbiotics produced from *L. rhamnosus* had an inhibitory impact on the expression of NF-kB in renal epithelial cells, as well as other oxidative indicators, including Caspase-3, p53, and ERK, which is in line with prior research on probiotic fermented supernatants^59^. We hypothesized that the postbiotics MSD-21, MSD-24, and MSD-37, associated with isoflavonoid compounds (**1**–**7**) isolated from *Iris albicans* or its extract, exhibit anti-aging effects by inhibiting oxidative markers linked to the AGE-RAGE interaction.

The Prebiotic Activity Score (A_preb_) allows an accurate quantification of the ability of a substance to stimulate the growth or activity of probiotics compared with pathogenic bacteria. This score is an excellent metric for assessing possible prebiotics and understanding their working mechanism for gut health^11–13^. To our knowledge, no previous research has evaluated the prebiotic activity score of postbiotics. Although postbiotics are not traditionally classified as prebiotics, they can exhibit prebiotic-like effects in specific contexts. This is because postbiotics, which consist of metabolites, cell wall components, and other bioactive substances derived from probiotic microorganisms, can modulate the composition and activity of gut microbiota in ways like prebiotics. In this study, we specifically investigated the effects of postbiotics produced using seven individual isoflavonoids or *Iris albicans* extract as carbon sources on the activation of various probiotic strains, comparing them to postbiotics derived from glucose as a carbon source. The findings revealed that postbiotics derived from isoflavonoid compounds or *Iris albicans* extract outperformed the control (glucose-derived postbiotics) in terms of their activity. Our research team effectively used pure phenolic compounds derived from select plants, in addition to extracts of these plants, as carbon source media and screening for multiple probiotic strains in some previous studies. All presented findings were encouraging^11–13,60^, since phenolic compounds and flavonoids are comprised of added definitions regarding prebiotics in the newly revised standard for prebiotics^61^. Similar enzymatic profiles of *Lactobacillus*spp. strains have been described by Elbermawi, et al^12^. It was noted that N-acetyl-β-glucosaminidase, alkaline phosphatase, and cystine arylamidase activity were carbohydrate-dependent. *β*-Sitosterol 3-*O*-*β*-D-glucoside, 6`-*O*-(4``-hydroxy-trans-cinnamoyl)-kaempferol-3-*O*-*β*-D-glucopyranoside, tamarixetin-3-*O*-*β*-D-glucoside and myricetin-3-*O*-α-L-rhamnosid inhibited N-acetyl-*β*-glucosaminidase activity in *Lactobacillus* spp. strains, which indicates a favorable change in metabolic profile, because the enzyme could cause intestinal diseases. These compounds, used as the carbon source, also resulted in a promotion of alkaline phosphatase activity, which constitutes a positive effect in terms of probiotic properties of the strains, since this enzyme could improve immunomodulation and inhibit inflammatory responses in the GIT. In summary, replacing glucose with isoflavonoid compounds led to beneficial metabolic changes and enhanced some probiotic features of *Lactobacillus* spp. strains.

A key conceptual point emerging from our data is the relative contribution of fermentation‑driven transformation versus the intrinsic activity of the carbon source. Although the present study did not include a direct comparison between non‑fermented carbon sources (e.g., IR extract or individual compounds in uninoculated medium) and their corresponding postbiotic preparations under identical assay conditions, several observations support a genuine added value of the postbiotic preparation process. In particular, the pronounced strain‑dependent differences observed among MSD‑21, MSD‑24, and MSD‑37 when grown on the same carbon source (including IR extract) across antioxidant, antiglycation, and prebiotic assays indicate that microbial metabolism and the resulting postbiotic metabolite profiles, rather than the substrate alone, are critical determinants of bioactivity. Collectively, these findings suggest that the fermentation step converts the original phytochemical substrates into distinct postbiotic metabolite signatures that underpin the enhanced functional properties of the preparations, while we acknowledge that future work should include side‑by‑side testing of non‑fermented substrates versus their corresponding postbiotics to quantitatively disentangle substrate‑intrinsic from fermentation‑derived effects.

Although valuable findings on the antioxidant and anti‑AGEs activities of the isoflavonoid‑derived postbiotics have been demonstrated, the study’s outcomes face several challenges when applied in vivo. Stability and bioavailability studies of postbiotics in living organisms are still needed. More research is necessary to determine the ability of postbiotics to replace true prebiotics, their impact on gut flora, and their role in therapeutic applications for health improvement. In addition, aminoguanidine was used only as a fixed‑dose positive control, and the complex nature of the postbiotic preparations (mixtures of residual substrates and fermentation‑derived metabolites) did not allow us to calculate a precise equivalent concentration or IC_50_-type potency relative to aminoguanidine; future studies should therefore include dose–response experiments and standardization of postbiotic preparations to enable meaningful equivalent‑concentration comparisons with this reference inhibitor. Finally, we did not perform systematic comparisons with non‑fermented substrates (IR extract or individual compounds in un‑inoculated medium), so the present data cannot fully disentangle substrate‑intrinsic effects from those generated during fermentation; follow‑up work should incorporate side‑by‑side testing of fermented versus non‑fermented preparations to better quantify the added value of the postbiotic production process.

## Conclusions

The Postbiotics in the current study were derived from seven individual isoflavonoid compounds extracted from I. albicans or its methanolic extract, serving as the carbon source. It was found that these postbiotics were functionally beneficial by reducing advanced glycation end-products (AGEs) and fructosamine levels, inhibiting protein oxidation and aggregation, and diminishing binding of AGEs to RAGE. Such postbiotics (*L. fermentum* MSD24, *L. casei* MSD21, *L. reuteri* MSD37) also increased antioxidant activity (DPPH, ABTS, and FRAP assays), amounts of polyphenols, as well as free thiol and amino group availability, and the prebiotic activity scores of selected probiotic strains. Such findings validate the potential of *I. albicans* as a natural and effective source for bioactive compounds that manage age-related diseases. However, more in vivo studies tend to require medical validation of these encouraging findings.

## Supplementary Information

Below is the link to the electronic supplementary material.

Supplementary Material 1

## Data Availability

All data generated or analyzed during this study are available from the corresponding author on reasonable request.
