# Supplementary material for: Isoflavonoids from Iris albicans as a carbon source to enhance the anti-aging potential of lactic acid bacteria-derived postbiotics
Source: Sci Rep. 2026 Jun 4;16:17348. doi: 10.1038/s41598-026-52823-x (PMC13237060; doi:10.1038/s41598-026-52823-x)
Supplement: Supplementary file 1 — Supplementary Material 1 [file 41598_2026_52823_MOESM1_ESM.docx]

**Isoflavonoids from *Iris albicans* as a Carbon Source to Enhance the Anti-Aging Potential of Lactic Acid Bacteria-Derived Postbiotics**

**Ahmed Elbermawi^1^, Mohamed Samir Darwish^2*^, Noha A. Abou-Zeid^3^, Ahmed A. Zaki^1*^, Asmaa A. Elawady^2^, and Yhiya Amen^1^**

^1^ Department of Pharmacognosy, Faculty of Pharmacy, Mansoura University, Mansoura, 35516 Egypt, asbeder@mans.edu.eg (A.E.), ahmed.awad@fulbrightmail.org (A.A.Z.), yhiaamen@mans.edu.eg (Y.A.).

^2^ Dairy Department, Faculty of Agriculture, Mansoura University, Mansoura 35516, Egypt, asmaa-elawady@mans.edu.eg (A.A.E).

^3^ Veterinary Medicine Directorate, Mansoura 35516, Egypt, nanoodr@gmail.com (N.A.A.).

***** Correspondence: [msamir@mans.edu.eg](mailto:msamir@mans.edu.eg) (M.S.D), [ahmed.awad@fulbrightmail.org](mailto:ahmed.awad@fulbrightmail.org) (A.A.Z.)

**List of contents**

| **Figure S1** | HRESI^+^MS [M+H] spectrum of compound **1** | **2** |
| --- | --- | --- |
| **Figure S2** | ^1^H-NMR spectrum of compound **1** | **2** |
| **Figure S3** | ^13^C-NMR spectrum of compound **1** | **3** |
| **Figure S4** | HRESI^+^MS [M+Na] ^+^ of compound **2** | **3** |
| **Figure S5** | ^1^H-NMR spectrum of compound **2** | **4** |
| **Figure S6** | ^13^C-NMR spectrum of compound **2** | **4** |
| **Figure S7** | HRESI^-^MS [M-H] of compound **3** | **5** |
| **Figure S8** | ^1^H-NMR spectrum of compound **3** | **5** |
| **Figure S9** | ^13^C-NMR spectrum of compound **3** | **6** |
| **Figure S10** | HRESI^+^MS [M+H] of compound **4** | **6** |
| **Figure S11** | ^1^H-NMR spectrum of compound **4** | **7** |
| **Figure S12** | ^13^C-NMR spectrum of compound **4** | **7** |
| **Figure S13** | HRESI^-^MS [M-H] spectrum of compound **5** | **8** |
| **Figure S14** | ^1^H-NMR spectrum of compound **5** | **8** |
| **Figure S15** | ^13^C-NMR spectrum of compound **5** | **9** |
| **Figure S16** | HRESI^+^MS [M+K] spectrum of compound **6** | **9** |
| **Figure S17** | ^1^H-NMR spectrum of compound **6** | **10** |
| **Figure S18** | ^13^C-NMR spectrum of compound **6** | **10** |
| **Figure S19** | HRESI^+^MS [M+H] spectrum of compound **7** | **11** |
| **Figure S20** | ^1^H-NMR spectrum of compound **7** | **11** |
| **Figure S21** | ^13^C-NMR spectrum of compound **7** | **12** |
| **Table S1** | Factor loadings | **15** |
| **Table S2** | Correlations between variables and factors | **16** |

**Materials and Methods**

- 1. **Chemicals**

Ammonium citrate, anhydrous sodium acetate, magnesium sulfate, manganese sulfate, dipotassium phosphate, 2,2-diphenyl-1-picrylhydrazyl radical (DPPH·), APTS solution, dimethyl sulfoxide (DMSO), ferric chloride, 2,4,6-tris(2-pyridyl)-s-triazine (TPTZ), hydrochloric acid (HCl), aminoguanidine, gallic acid, bovine serum albumin (BSA), fructose, sodium azide, nitroblue tetrazolium (NBT), 1-deoxy-1-morpholinofructose (1-DMF), 2,4,6-trinitrobenzene sulfonic acid, sodium dodecyl sulfate (SDS), 2,4-dinitrophenylhydrazine (DNPH), ethanol, ethyl acetate, 5,5′-dithiobis(2-nitrobenzoic acid) (DTNB), and thioflavin T.

- 1. **Culture media and strains**

Bacterial strains (*Limosilactobacillus fermentum* MSD24, *Lacticaseibacillus casei* MSD21, *Limosilactobacillus reuteri* MSD37, *Lactiplantibacillus plantarum* MSD74, *Lacticaseibacillus rhamnosus* ML57, *Lacticaseibacillus paracasei* MSD108, *Escherichia coli* K12) were provided by the microbiology laboratory’s culture collection at the Dairy Department, Faculty of Agriculture, Mansoura University, Egypt. The bacterial culture media, including de Man, Rogosa, and Sharpe (MRS) broth, MRS agar, nutrient agar, and nutrient broth were obtained from Thermo Fisher Scientific in Cairo, Egypt. The API ZYM test kit was purchased from bioMérieux SA (Marcy l’Etoile, France).

- 1. **General Procedures**

^1^H and ^13^C-NMR spectra were obtained on a Bruker DRX 600 NMR spectrometer (Bruker Daltonics INC., MA, USA). Chemical shifts (δ) were expressed in ppm with reference to the TMS resonance. HR-ESI-MS was determined using LC–MS–IT–TOF (Shimadzu, Tokyo, Japan). The MS instrument was operated using an ESI source in both positive and negative ionization modes with survey scans acquired from *m/z* 100–2000 for MS and *m/z* 50–1500 for MS/MS. The ionization parameters were as follows: probe voltage, ±4.5 kV; nebulizer gas flow, 1.5 L/min; CDL temperature, 200 °C; heat block temperature, 200°C. Silica gel (75–120 mesh) was purchased from Merck (Cairo, Egypt). Thin-layer chromatography (TLC) silica gel 60 F_254_ was purchased from Merck (Cairo, Egypt). Sephadex LH-20 was purchased from GE Healthcare (Uppsala, Sweden). The developed chromatograms were visualized under 254 nm. UV light and the spots were made visible by spraying with a vanillin/H2SO4 reagent before warming in an oven preheated to 110 °C for 5 min.

- 1. **Extraction and Isolation**

The powdered rhizomes of *I. albicans* L. (305 g) were extracted with 70% methanol (6 × 2 L) at room temperature to get a dark brown residue (17.6 g). The total alcoholic extract was subjected to VLC over normal silica gel using CHCl3 with increasing proportions of MeOH to afford four fractions: 5% MeOH fraction (A, 2.6 g), 10% MeOH fraction (B, 4.0 g), 20% MeOH fraction (C, 3.2 g), and 30% MeOH (D, 5.3 g) fractions. Fraction A (2.6 g) was chromatographed over a silica gel column (2 × 50 cm) prepared in petroleum ether. The column was run with a pet. ether-EtOAc gradient from (100:0 to 50:50) to give 6 fractions (1-6). Fraction 1, which eluted from the column by pet. ether-EtOAc 15% was subjected to fine purification over Sephadex LH-20 eluted with 100% MeOH to get compound **1 (20.4 mg)** in pure form. Fraction 2, which eluted from the column by pet. ether : EtOAc (8 : 2), was purified after re-chromatography over normal preparative TLC using pet. ether: EtOAc (7 : 3) as a developing system to yield compound **2 (3.7 mg)**. Fraction 3, which eluted from the column by pet. ether: EtOAc (7.5: 2.5), was purified by crystallization after washing with diethyl ether: acetone 1:1 to yield compound **3 (21 mg)**. Fraction 4, which eluted from the column by pet. ether: ethyl acetate 30%, was subjected to fine purification over sephadex LH-20 eluted with 100% MeOH to get compound **4 (6.9 mg)**. Fraction 5, which was eluted from the column by pet. ether: ethyl acetate 35%, was re-chromatographed over sephadex LH-20 eluted with 100% MeOH to yield compound **5 (14.1 mg)**. Fraction 6 (523 mg), which was eluted from the column by pet. ether: EtOAc (6 : 4), was subjected to re-chromatography using a silica gel column eluted with a pet. ether: EtOAc gradient from (8: 2 to 6: 4) to give 3 subfractions (6a-5c). Subfraction 6c (100 mg), which was eluted from the column by pet. ether: ethyl acetate 50%, was re-chromatographed over Sephadex LH-20 eluted with 100% MeOH to yield compound **6 (5.9 mg)**. Fraction C (3.2 g) was chromatographed over a silica gel column (3 × 50 cm) prepared in petroleum ether. The column was run with a petroleum ether : EtOAc gradient from (1 :1 to 0:1) to give 4 subfractions (7-10). Subfraction 10 (1.4 g), which was eluted from the column by 100% EtOAc, was subjected to fine purification over Sephadex LH-20 eluted with 100% MeOH to obtain compound **7 (6.5 mg)**.


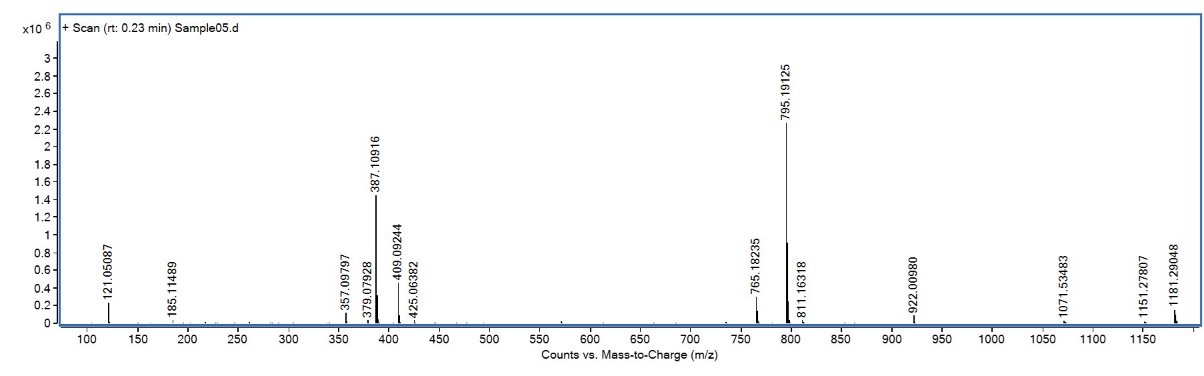


Figure S1: HRESI^+^MS [M+H] spectrum of compound **1**


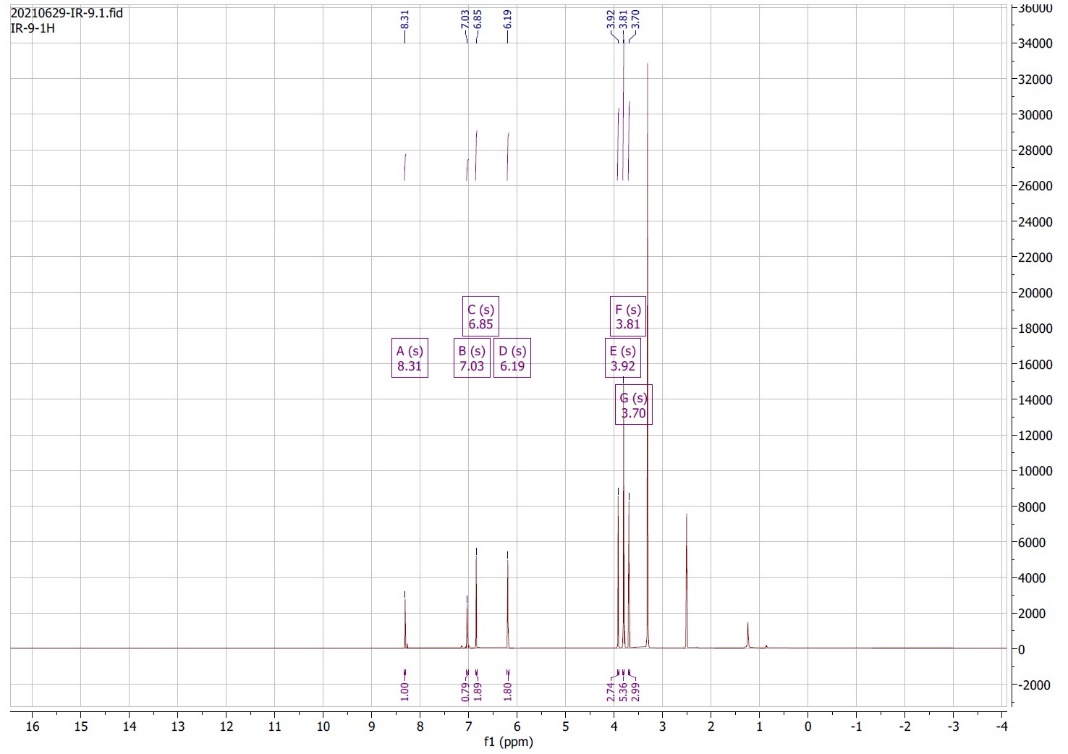


Figure S2: ^1^H-NMR spectrum of compound **1**


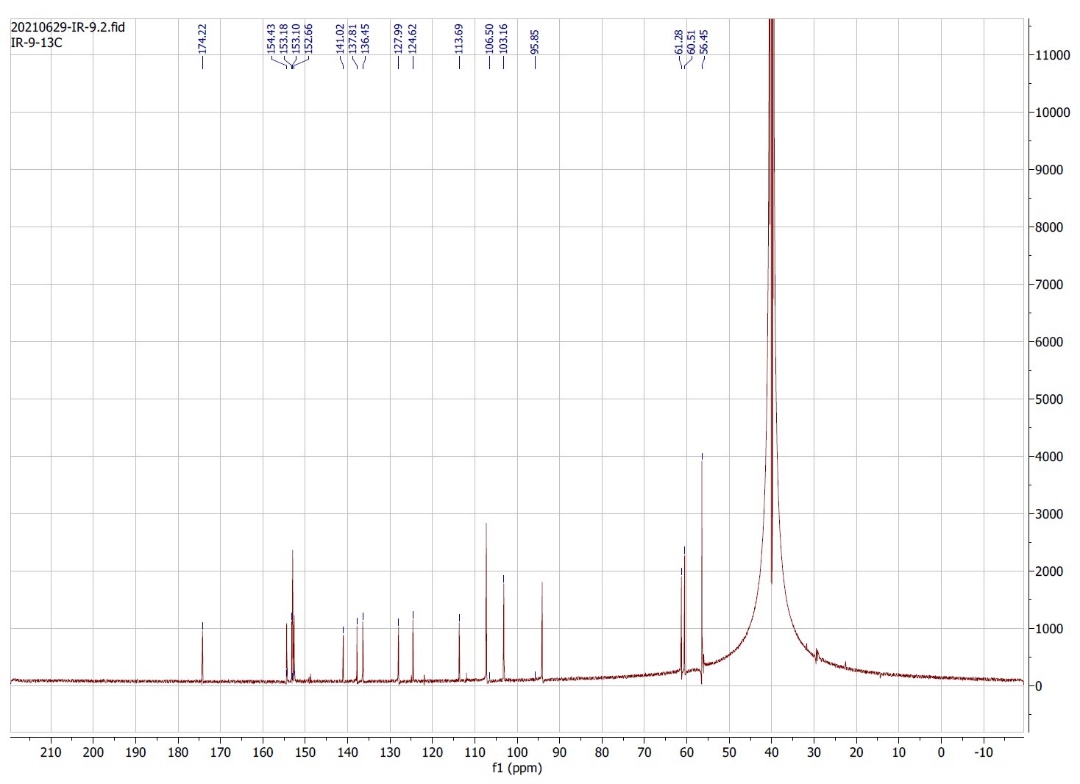


Figure S3: ^13^C-NMR spectrum of compound 1


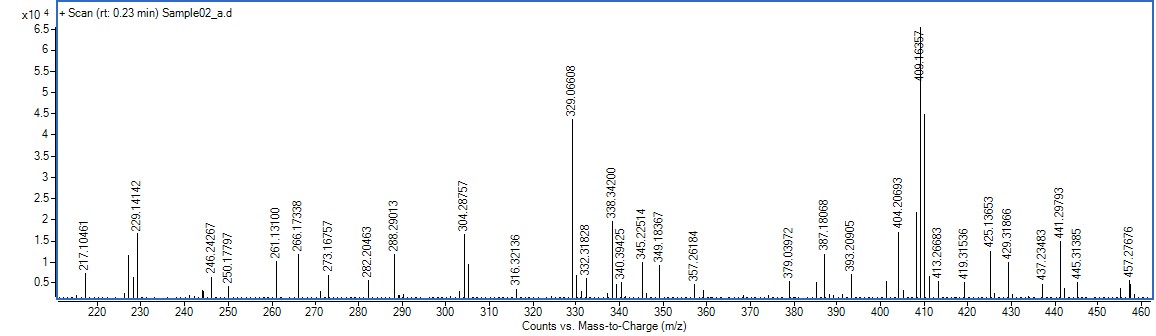


Figure S4: HRESI^+^MS [M+Na] ^+^ of compound **2**


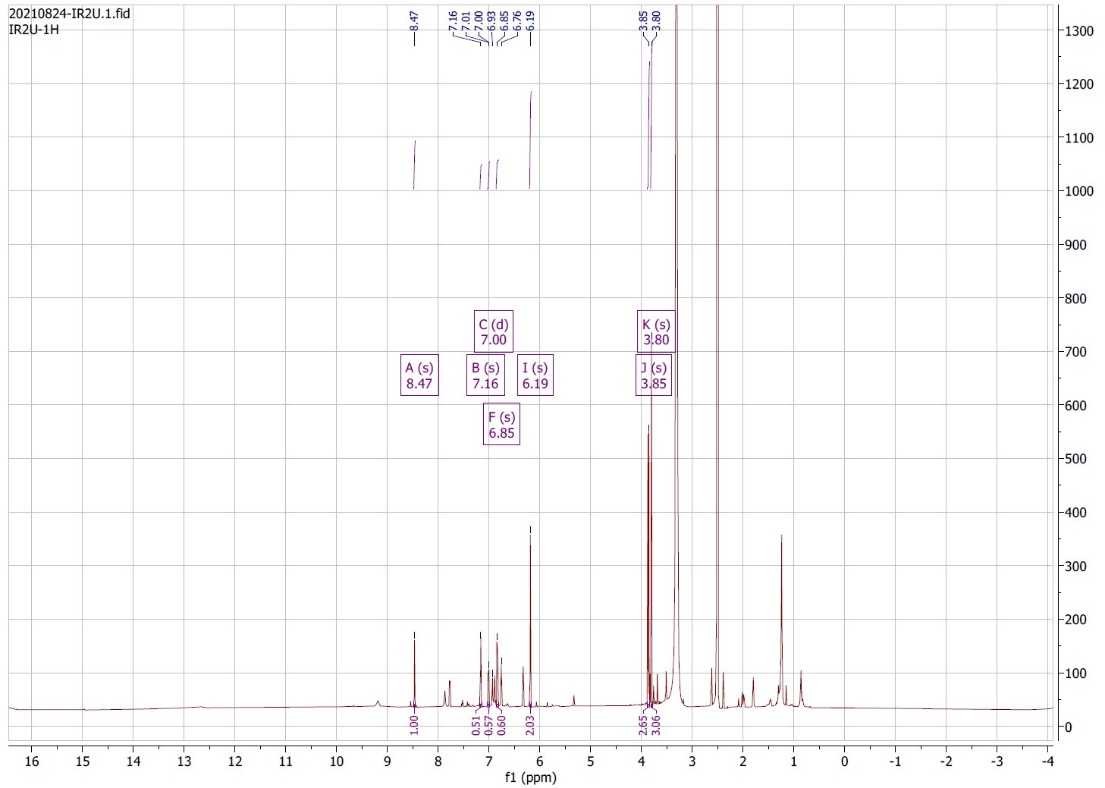


Figure S5: ^1^H-NMR spectrum of compound **2**


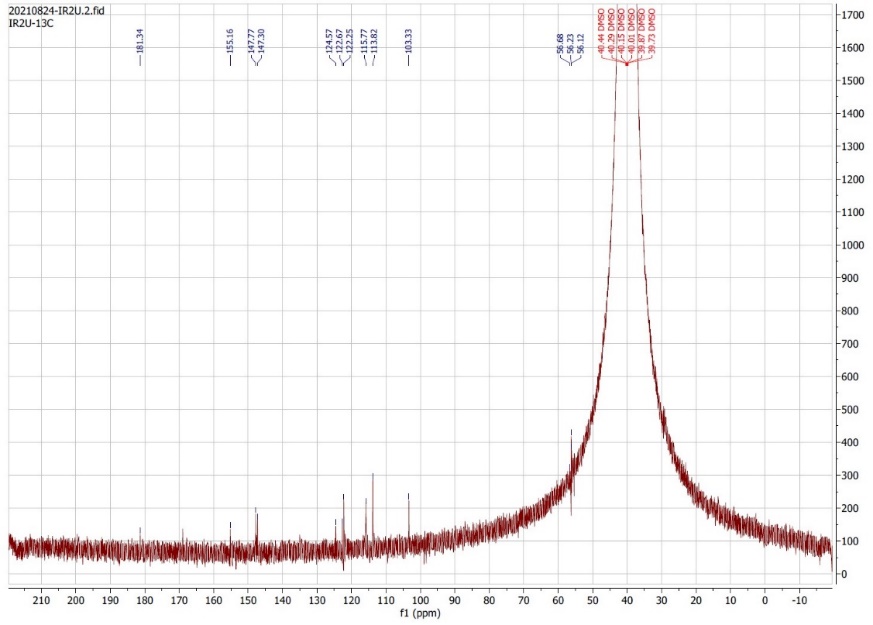


Figure S6: ^13^C-NMR spectrum of compound **2**


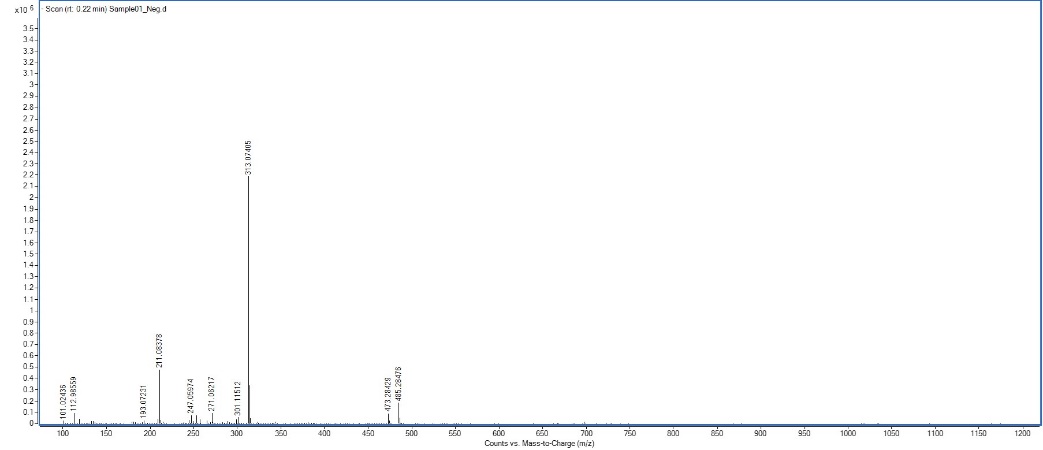


Figure S7: HRESI^-^MS [M-H] of compound **3**


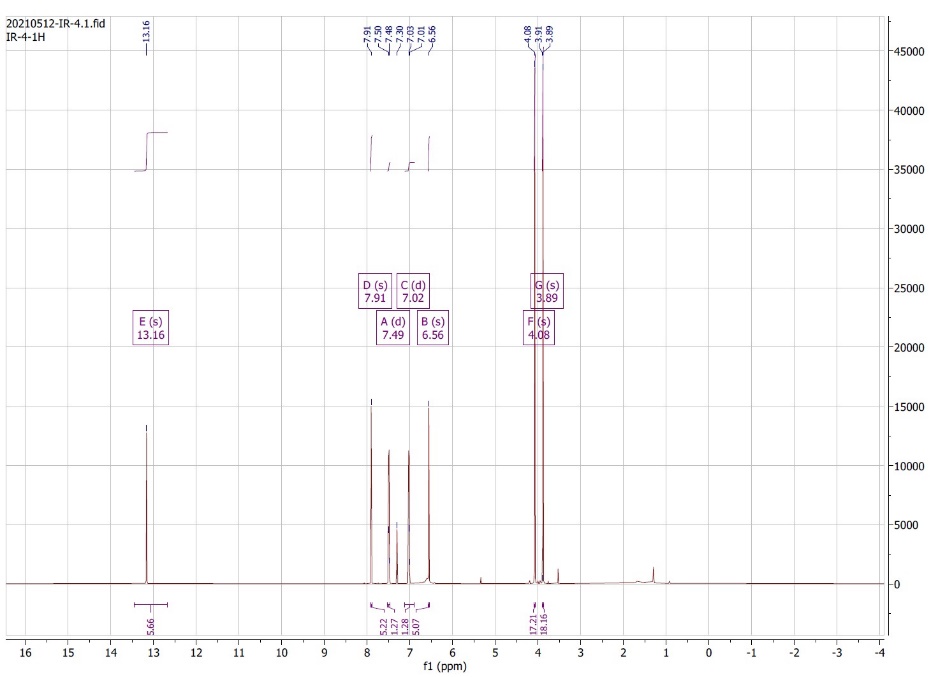


Figure S8: ^1^H-NMR spectrum of compound **3**


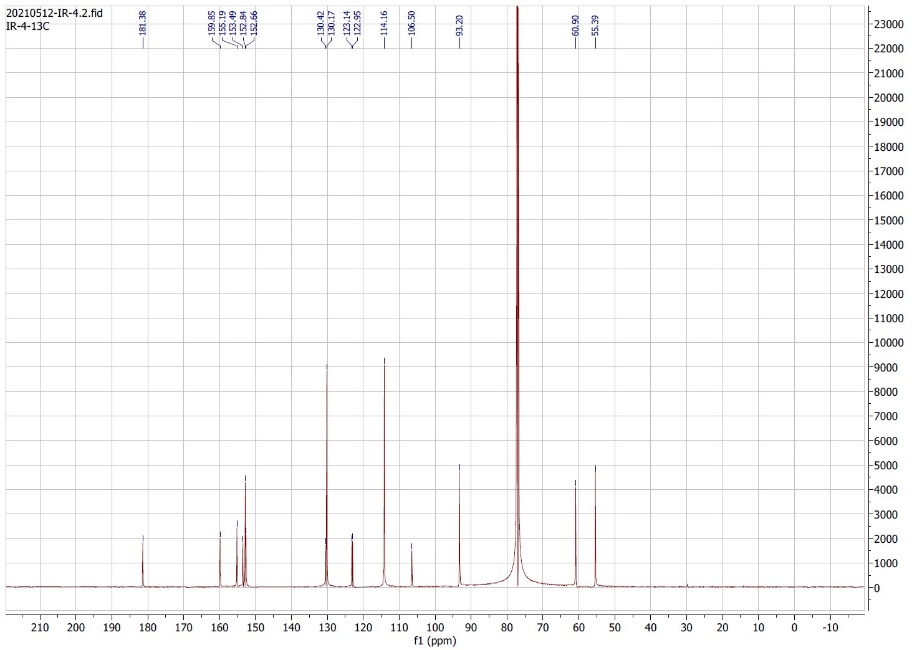


Figure S9: ^13^C-NMR spectrum of compound **3**


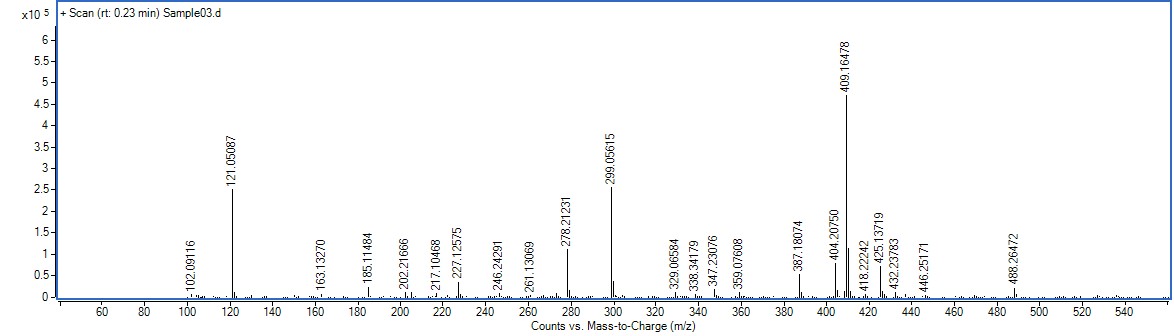


Figure S10: HRESI^+^MS [M+H] of compound **4**


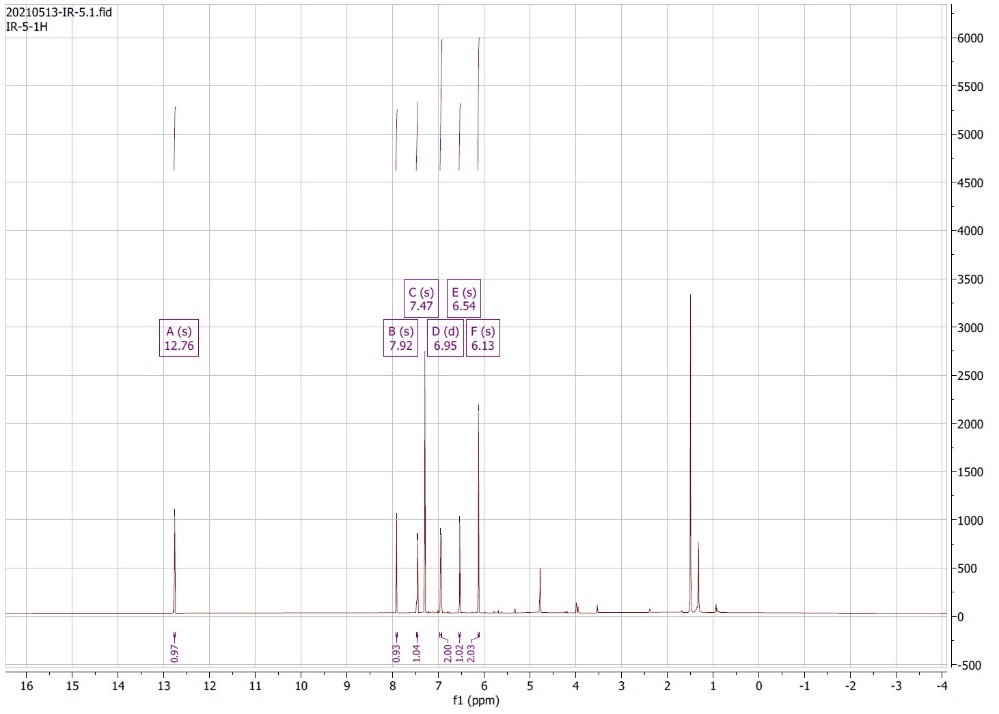


Figure S11: ^1^H-NMR spectrum of compound **4**


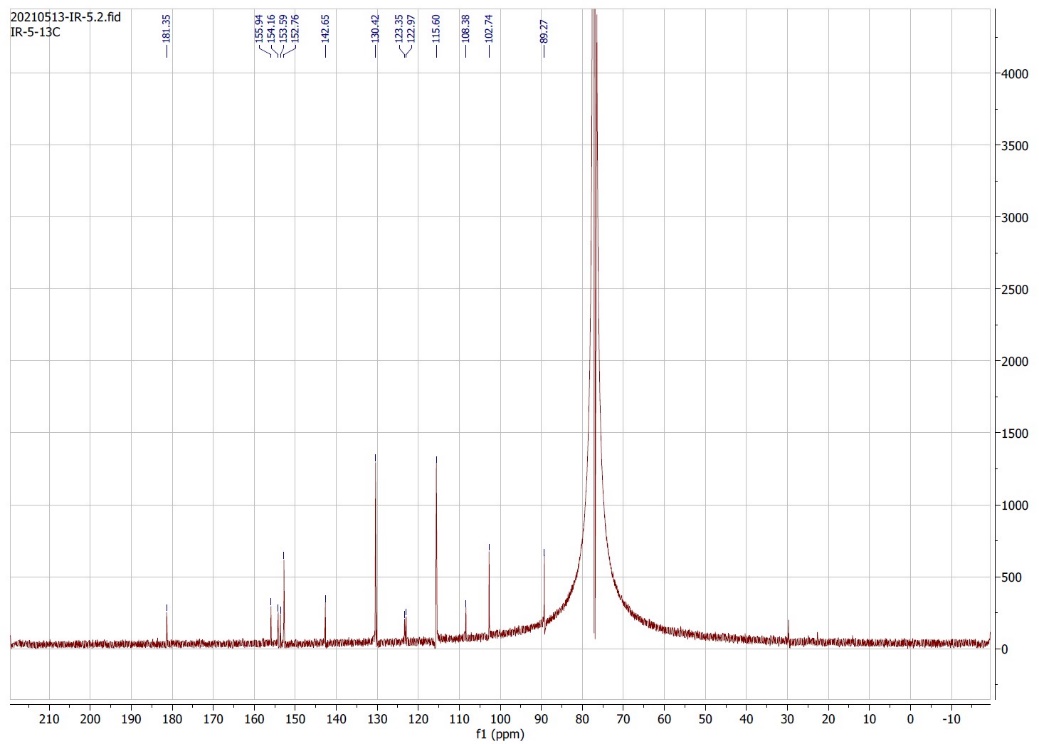


Figure S12: ^13^C-NMR spectrum of compound **4**


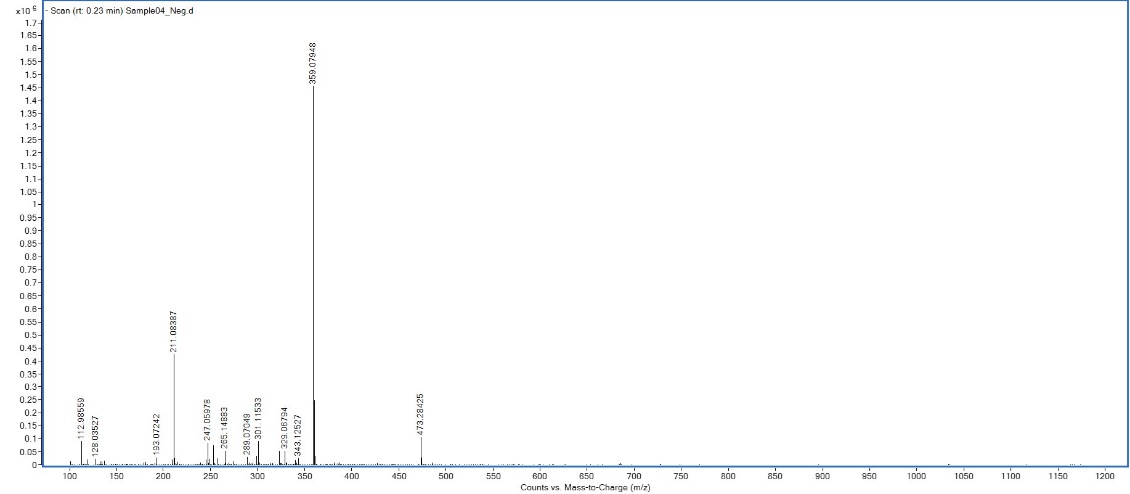


Figure S13: HRESI^-^MS [M-H] spectrum of compound **5**


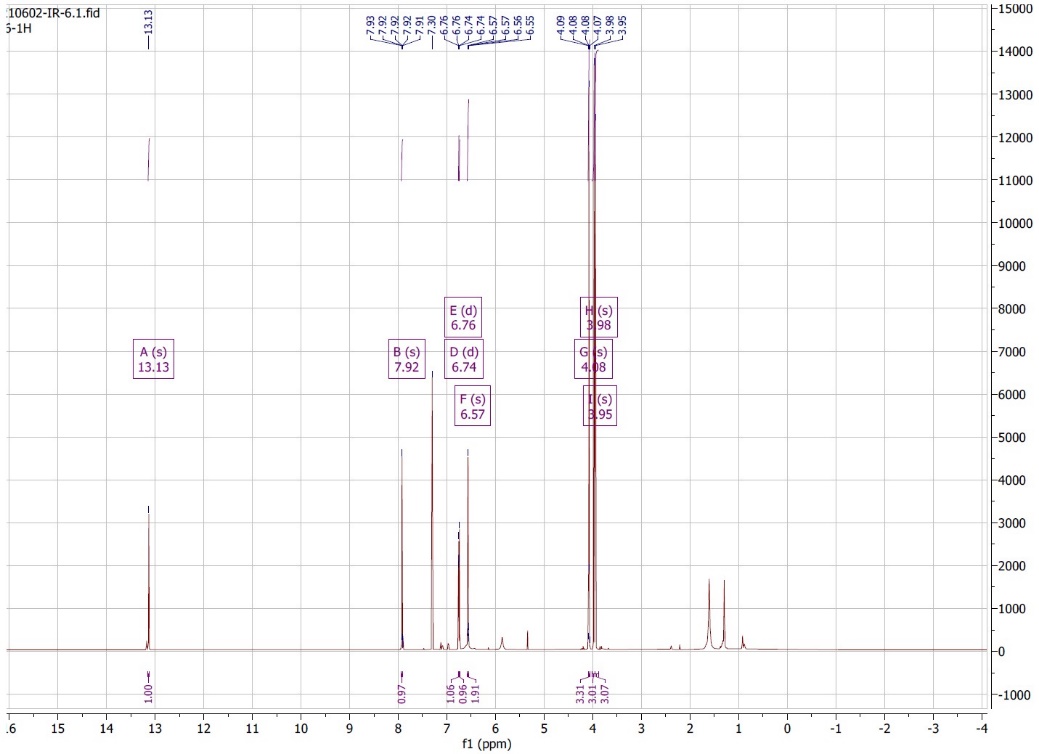


Figure S14: ^1^H-NMR spectrum of compound **5**


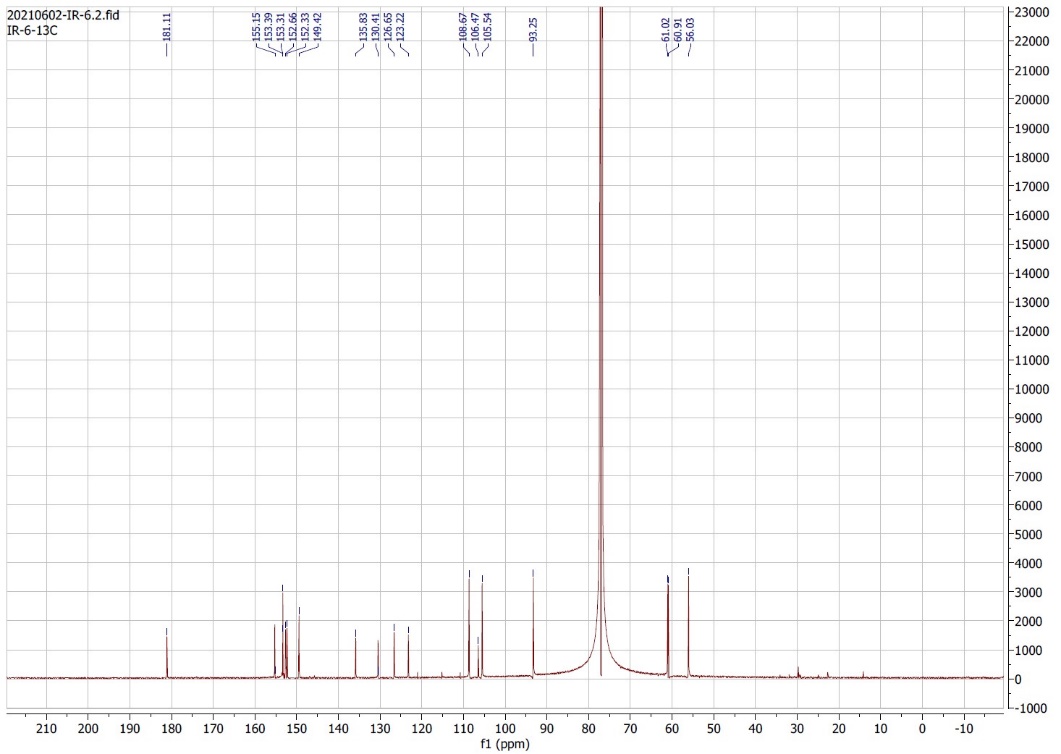


Figure S15: ^13^C-NMR spectrum of compound **5**


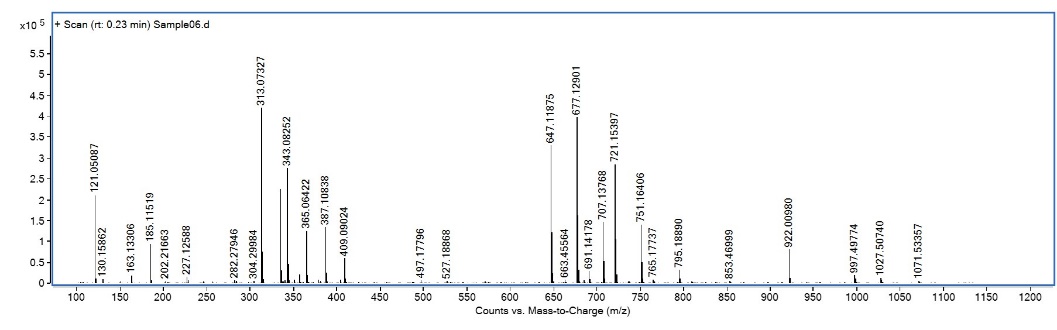


Figure S16: HRESI^+^MS [M+K] spectrum of compound **6**


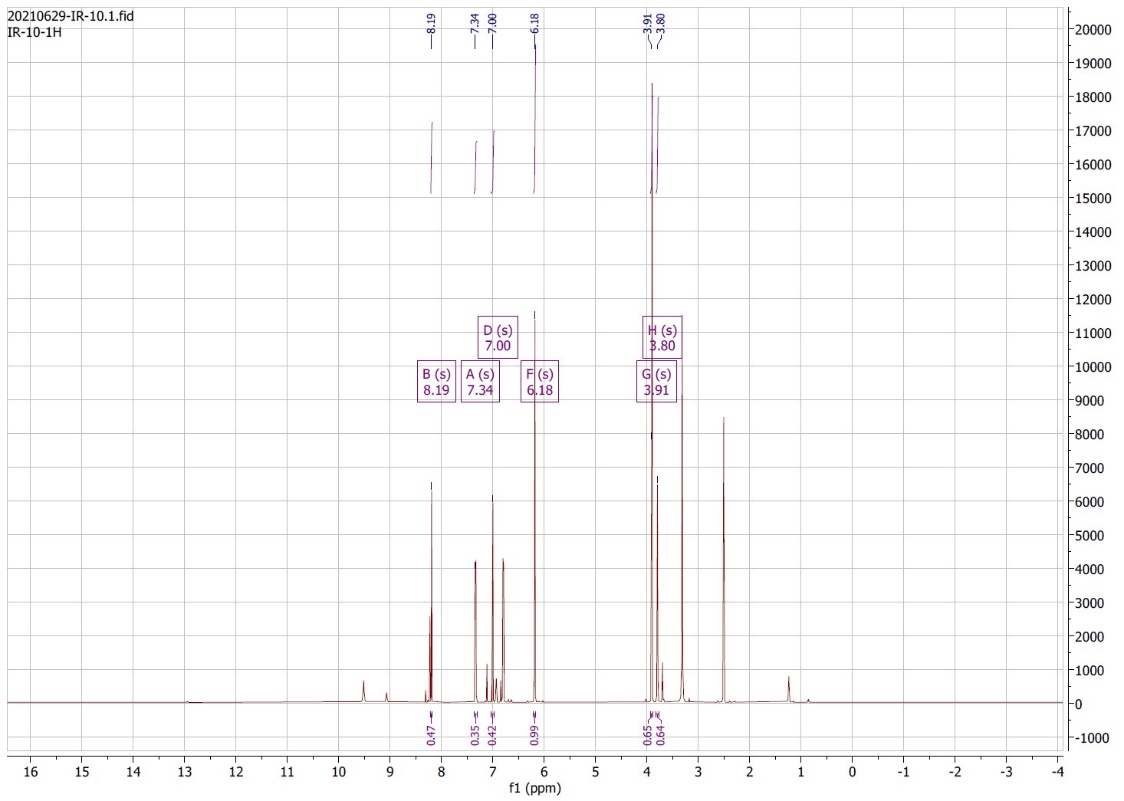


Figure S17: ^1^H-NMR spectrum of compound **6**


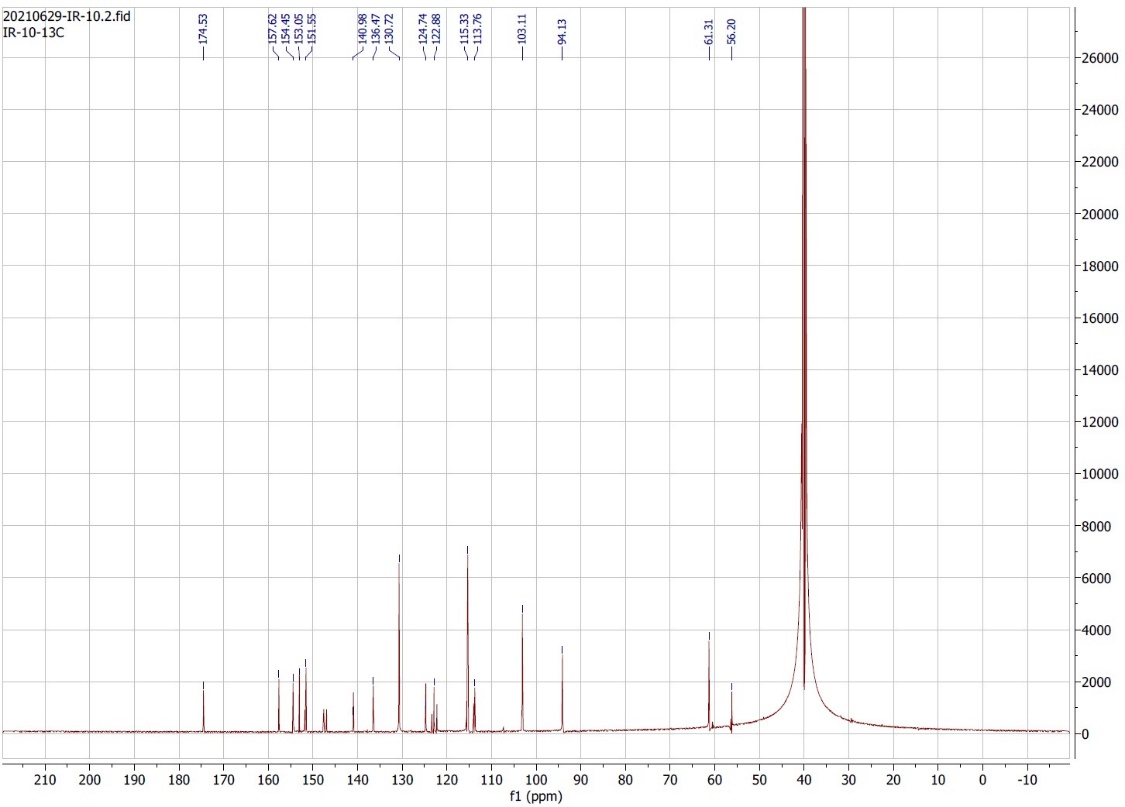


Figure S18: ^13^C-NMR spectrum of compound **6**


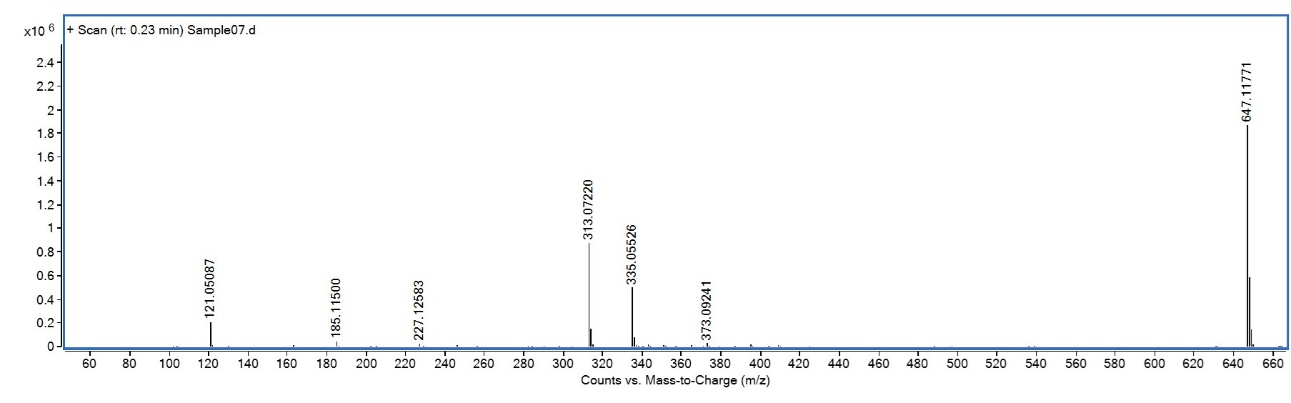


Figure S19: HRESI^+^MS [M+H] spectrum of compound **7**


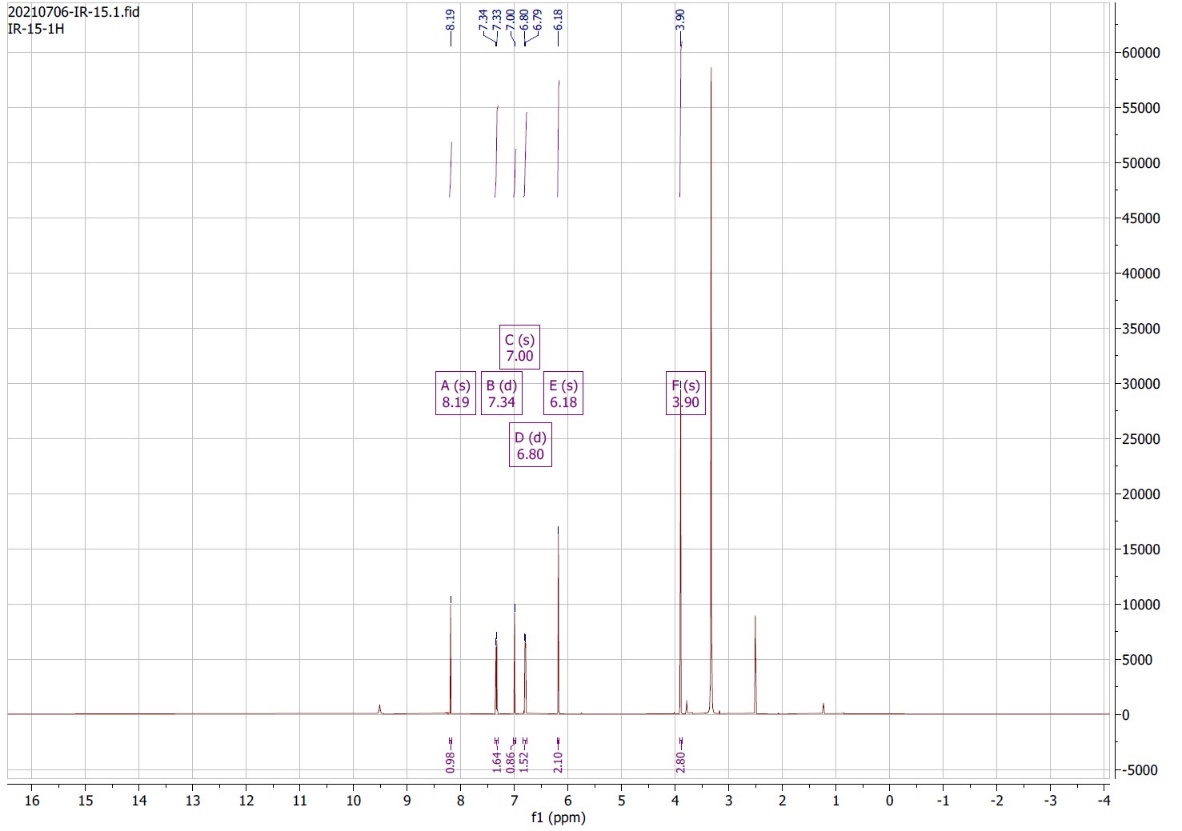


Figure S20: ^1^H-NMR spectrum of compound **7**


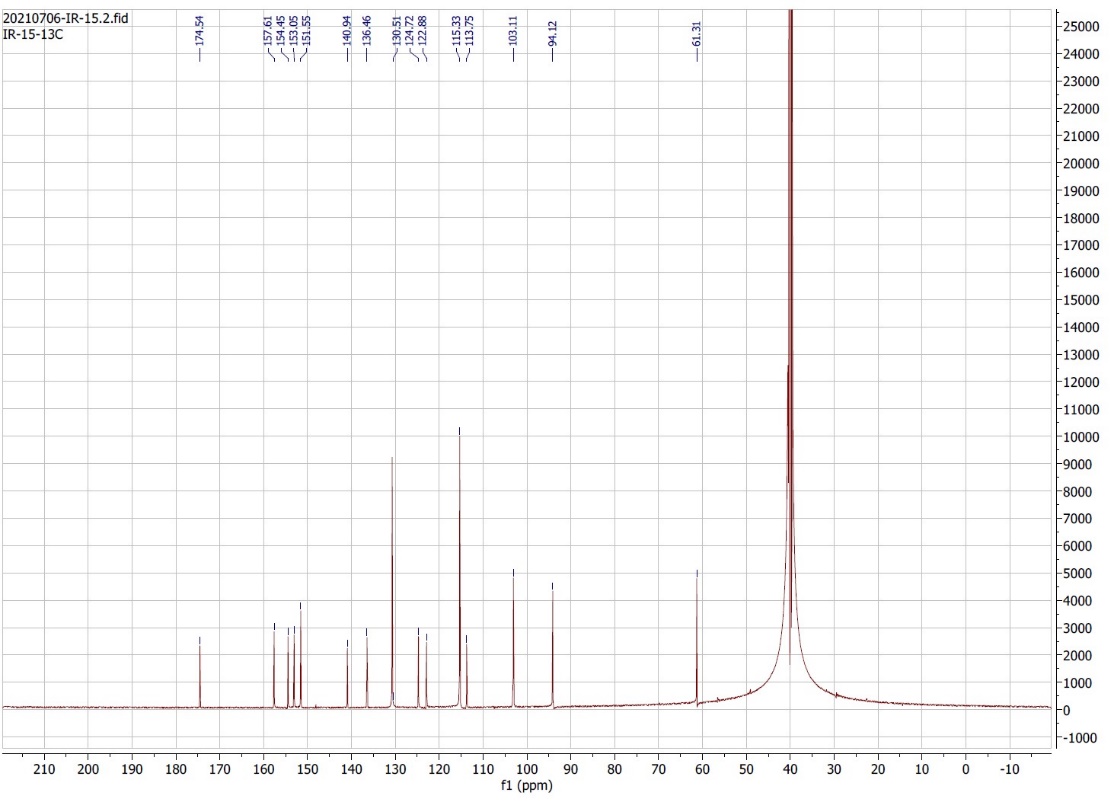


Figure S21: ^13^C-NMR spectrum of compound **7**

Table S1: Factor loadings

|  | F1 | F2 | F3 | F4 | F5 | F6 | F7 | F8 | F9 | F10 | F11 | F12 | F13 | F14 |
| --- | --- | --- | --- | --- | --- | --- | --- | --- | --- | --- | --- | --- | --- | --- |
| DPPH | 0.957 | -0.150 | 0.182 | -0.036 | 0.131 | 0.043 | 0.040 | -0.067 | -0.018 | -0.006 | -0.014 | 0.031 | 0.008 | 0.002 |
| ABTS | 0.951 | -0.082 | 0.195 | -0.177 | 0.131 | -0.009 | -0.013 | 0.010 | -0.022 | -0.024 | 0.038 | -0.015 | 0.000 | -0.001 |
| FRAP | 0.984 | -0.006 | 0.066 | -0.130 | 0.051 | 0.034 | 0.069 | -0.036 | 0.013 | 0.010 | -0.026 | -0.015 | -0.013 | -0.009 |
| Polyphenols | 0.606 | 0.679 | 0.400 | 0.097 | 0.002 | -0.028 | -0.018 | -0.005 | 0.003 | 0.006 | 0.001 | 0.001 | 0.001 | 0.000 |
| AGEs | 0.942 | -0.206 | 0.214 | -0.017 | 0.002 | 0.110 | -0.088 | 0.042 | 0.051 | 0.001 | -0.007 | -0.004 | 0.005 | 0.004 |
| Ketosamine | -0.860 | 0.340 | -0.265 | -0.198 | 0.074 | 0.165 | -0.041 | -0.036 | 0.001 | 0.013 | 0.009 | 0.004 | 0.002 | 0.000 |
| Carbonyl | -0.964 | 0.079 | -0.021 | -0.132 | 0.156 | -0.134 | 0.007 | -0.054 | 0.033 | -0.014 | -0.012 | -0.008 | 0.002 | 0.008 |
| Amino group | -0.936 | 0.015 | 0.010 | 0.261 | 0.196 | 0.101 | 0.078 | 0.017 | 0.016 | -0.023 | 0.007 | -0.005 | 0.000 | -0.002 |
| SH | 0.951 | 0.135 | -0.148 | -0.067 | -0.188 | 0.016 | 0.115 | -0.012 | 0.034 | -0.020 | 0.020 | 0.009 | 0.000 | 0.003 |
| Thioflavin T | -0.965 | 0.082 | 0.104 | -0.188 | 0.011 | -0.029 | 0.018 | 0.119 | 0.005 | -0.018 | -0.013 | 0.016 | 0.003 | -0.007 |
| RAGE | 0.953 | 0.128 | -0.267 | 0.042 | -0.014 | -0.014 | -0.023 | -0.009 | -0.008 | -0.022 | -0.018 | -0.014 | 0.022 | -0.005 |
| MSD-74 | 0.946 | 0.077 | -0.273 | 0.074 | 0.076 | -0.047 | -0.102 | -0.007 | 0.012 | -0.025 | 0.002 | 0.017 | -0.015 | -0.004 |
| ML-57 | 0.967 | 0.149 | -0.167 | -0.016 | 0.062 | 0.045 | 0.035 | 0.082 | -0.026 | -0.005 | -0.019 | -0.006 | -0.008 | 0.011 |
| MSD-108 | 0.957 | 0.004 | -0.200 | 0.025 | 0.173 | -0.078 | 0.038 | 0.051 | 0.017 | 0.043 | 0.016 | 0.006 | 0.007 | -0.002 |

Table S2: Correlations between variables and factors

|  | F1 | F2 | F3 | F4 | F5 | F6 | F7 | F8 | F9 | F10 | F11 | F12 | F13 | F14 |
| --- | --- | --- | --- | --- | --- | --- | --- | --- | --- | --- | --- | --- | --- | --- |
| DPPH | 0.957 | -0.150 | 0.182 | -0.036 | 0.131 | 0.043 | 0.040 | -0.067 | -0.018 | -0.006 | -0.014 | 0.031 | 0.008 | 0.002 |
| ABTS | 0.951 | -0.082 | 0.195 | -0.177 | 0.131 | -0.009 | -0.013 | 0.010 | -0.022 | -0.024 | 0.038 | -0.015 | 0.000 | -0.001 |
| FRAP | 0.984 | -0.006 | 0.066 | -0.130 | 0.051 | 0.034 | 0.069 | -0.036 | 0.013 | 0.010 | -0.026 | -0.015 | -0.013 | -0.009 |
| Polyphenols | 0.606 | 0.679 | 0.400 | 0.097 | 0.002 | -0.028 | -0.018 | -0.005 | 0.003 | 0.006 | 0.001 | 0.001 | 0.001 | 0.000 |
| AGEs | 0.942 | -0.206 | 0.214 | -0.017 | 0.002 | 0.110 | -0.088 | 0.042 | 0.051 | 0.001 | -0.007 | -0.004 | 0.005 | 0.004 |
| Ketosamine | -0.860 | 0.340 | -0.265 | -0.198 | 0.074 | 0.165 | -0.041 | -0.036 | 0.001 | 0.013 | 0.009 | 0.004 | 0.002 | 0.000 |
| Carbonyl | -0.964 | 0.079 | -0.021 | -0.132 | 0.156 | -0.134 | 0.007 | -0.054 | 0.033 | -0.014 | -0.012 | -0.008 | 0.002 | 0.008 |
| Amino group | -0.936 | 0.015 | 0.010 | 0.261 | 0.196 | 0.101 | 0.078 | 0.017 | 0.016 | -0.023 | 0.007 | -0.005 | 0.000 | -0.002 |
| SH | 0.951 | 0.135 | -0.148 | -0.067 | -0.188 | 0.016 | 0.115 | -0.012 | 0.034 | -0.020 | 0.020 | 0.009 | 0.000 | 0.003 |
| Thioflavin T | -0.965 | 0.082 | 0.104 | -0.188 | 0.011 | -0.029 | 0.018 | 0.119 | 0.005 | -0.018 | -0.013 | 0.016 | 0.003 | -0.007 |
| RAGE | 0.953 | 0.128 | -0.267 | 0.042 | -0.014 | -0.014 | -0.023 | -0.009 | -0.008 | -0.022 | -0.018 | -0.014 | 0.022 | -0.005 |
| MSD-74 | 0.946 | 0.077 | -0.273 | 0.074 | 0.076 | -0.047 | -0.102 | -0.007 | 0.012 | -0.025 | 0.002 | 0.017 | -0.015 | -0.004 |
| ML-57 | 0.967 | 0.149 | -0.167 | -0.016 | 0.062 | 0.045 | 0.035 | 0.082 | -0.026 | -0.005 | -0.019 | -0.006 | -0.008 | 0.011 |
| MSD-108 | 0.957 | 0.004 | -0.200 | 0.025 | 0.173 | -0.078 | 0.038 | 0.051 | 0.017 | 0.043 | 0.016 | 0.006 | 0.007 | -0.002 |
